# Pan-cancer mutational signature analysis of 111,711 targeted sequenced tumors using SATS

**DOI:** 10.1101/2023.05.18.23290188

**Published:** 2024-07-31

**Authors:** Donghyuk Lee, Min Hua, Difei Wang, Lei Song, Tongwu Zhang, Xing Hua, Kai Yu, Xiaohong R. Yang, Stephen J. Chanock, Jianxin Shi, Maria Teresa Landi, Bin Zhu

## Abstract

Tumor mutational signatures are informative for cancer diagnosis and treatment. However, targeted sequencing, commonly used in clinical settings, lacks specialized analytical tools and a dedicated catalogue of mutational signatures. Here, we introduce SATS, a scalable mutational signature analyzer for targeted sequencing data. SATS leverages tumor mutational burdens to identify and quantify signatures in individual tumors, overcoming the challenges of sparse mutations and variable gene panels. Validations across simulated data, pseudo-targeted sequencing data, and matched whole-genome and targeted sequencing samples show that SATS can accurately detect common mutational signatures and estimate their burdens. Applying SATS to 111,711 tumors from the AACR Project GENIE, we created a pan-cancer mutational signature catalogue specific to targeted sequencing. We further validated signatures in lung, breast and colorectal cancers using an additional 16,774 independent samples. This signature catalogue is a valuable resource for estimating signature burdens in individual targeted sequenced tumors, facilitating the integration of mutational signatures with clinical data.

## Introduction

Tumors accumulate somatic mutations that form specific patterns, known as mutational signatures[1,2]. These signatures reveal the mutational processes driving carcinogenesis and can guide both the detection[3-5] and treatment[6-9] of cancer. To decipher these mutational signatures, multiple algorithms have been proposed[10-14], and catalogues of reference mutational signatures have been established for tumors analyzed through whole exome or whole genome sequencing (WES/WGS) in research settings[1,2].

Analyzing mutational signatures in clinical settings poses unique challenges due to the widespread use of diverse targeted sequencing panels. These panels, targeting cancer driver genes with therapeutic relevance, generate sparser mutation data compared to WES/WGS. Traditional *de novo* signature extraction[10-12] or signature refitting methods[13,14], optimized for WES/WGS, are not feasible to use with these limited data. In fact, *de novo* signature extraction requires a large number of samples with identical sequencing regions, which is rarely achievable with diverse targeted panels. Signature refitting, useful for limited samples (e.g., a single tumor), relies on pre-established reference signatures from WES/WGS data to estimate signature activities. The accuracy of this method depends on selecting reference signatures that are present in the sample, while excluding those that are not, to avoid misassigning mutations to irrelevant signatures[15]. However, the reference signatures found in WES/WGS tumors may miss signatures present in targeted sequenced tumors (e.g., treatment-induced) or may include rare signatures

NOTE: This preprint reports new research that has not been certified by peer review and should not be used to guide clinical practice.

unlikely to be present in targeted sequencing. Additionally, patient populations undergoing WES/WGS in research settings might differ from those undergoing targeted sequencing in real-world clinical settings, particularly for subjects under treatment, with rare cancer subtypes, or from underrepresented populations lacking WES/WGS data, which further complicates the application of traditional methods and WES/WGS-based reference signatures.

Recently, clustering methods have been proposed for detecting specific mutational signatures in targeted sequenced tumors. For example, the SigMA[16] algorithm is tailored to detect the HRD-associated signature SBS3, requiring pre-training with WGS data from individual tumors. However, this method detects just one signature, not multiple active mutational signatures simultaneously. The Mix[17] method, presents an alternative clustering strategy that does not rely on pre-training. However, it estimates signature activities at the cluster level rather than in individual samples. Hence, specialized analytical methods and a comprehensive catalogue of mutational signatures tailored to targeted sequenced tumors are needed.

Here, we introduce SATS (Signature Analyzer for Targeted Sequencing), a mutational signature analysis tool explicitly developed for targeted sequencing data. Unlike existing methods optimized for WES/WGS, SATS accounts for the variable size and genomic context of targeted gene panels and leverages large sample sizes of targeted sequencing studies. Our tests with simulated data, pseudo-targeted sequencing data generated by down-sampling whole exome and genome data, and matched WGS and targeted sequencing data showed that SATS outperforms other methods in detecting mutational signatures and estimating their burdens. Applying SATS, we established a pan-cancer catalogue of mutational signatures in 111,711 targeted sequenced tumors from a real-world clinico-genomic cancer registry, the AACR (American Association for Cancer Research) Project GENIE (Genomics Evidence Neoplasia Information Exchange, version 13.0-public, described below)[18,19]. Additionally, we validated the mutational signatures of lung, breast and colorectal cancers in an additional 13,425 samples from a newer version (15.1) of the AACR project GENIE, and confirmed signatures of lung and colorectal cancers in an independent Chinese cohort of 3,349 targeted sequenced tumors[20]. Finally, we show that through integration with clinical data, mutational signatures derived from targeted sequencing can identify the potential tissue of origin for tumors of unknown primary, find signatures enriched in early-onset hypermutated colorectal cancers, and serve as a biomarker for cancer immunotherapy response.

## Results

### Building a targeted sequencing-based mutational signature catalogue using SATS

SATS is developed for targeted sequencing to detect mutational signatures within a patient cohort and refit detected signatures to estimate signature burdens in individual patients. While we use single base substitutions (SBS) for the purpose of illustration, SATS is also adaptable to other types of somatic mutations, such as double base substitutions (DBS).

In analyzing SBS mutational signatures, SATS uses a mutation type matrix **V** as input that contains the counts of SBS across 96 mutation types within 32 trinucleotide contexts, such as a C to G mutation at the trinucleotide context TCT (i.e., a T[C>G]T mutation type). Additionally, SATS incorporates a panel context matrix **L** that specifies the number of trinucleotide contexts where a specific mutation type (e.g., TCT for T[C>G]T substitutions) could potentially occur in the targeted genes. SATS is based on a Poisson Nonnegative-Matrix Factorization (pNMF) model (Methods). The pNMF model decomposes the mutation type-by-patient matrix $\mathbf{V}$ into a mutation type-by-signature matrix $\mathbf{W}$ that describes signature profiles and a signature-by-patient matrix $\mathbf{H}$ that quantifies signature activities, while adjusting panel sizes by the panel context matrix $\mathbf{L}$ (Fig. 1a).

SATS comprises signature detection and signature refitting steps as outlined in Fig. 1b. In the signature detection phase, SATS first employs signeR[11] to discover *de novo* signatures from a targeted sequencing patient cohort. signeR is based on the pNMF model and adjusts for differences in the sizes of gene panels (Supplementary Note). Next, the *de novo* signature profiles are mapped to reference signatures from the pan-cancer COSMIC catalogue[1] using penalized nonnegative least squares (pNNLS)[21]. This allows to identify reference signatures that are present within the *de novo* signature profiles. Notably, these reference signatures can originate from any cancer type featured in the pan-cancer catalogue, not restricted to the specific cancer type of the patient cohort. During the signature refitting stage, we have developed an Expectation–Maximization (EM) algorithm, refitting the detected reference signatures to estimate signature activities in individual patients. Given that a variety of mutations can contribute to a mutational signature, we further estimate the expected number of mutations attributed to a reference signature, termed the signature burden, for each patient (Methods).

While direct detection of mutational signatures in a single patient is challenging, SATS can effectively estimate signature burdens at an individual level by adapting ("refitting") signatures previously identified in a large group of patients who have the same type of cancer and have been subjected to targeted sequencing. To enable this feature, we have compiled a pan-cancer catalogue of mutational signatures specific to targeted sequencing by analyzing 111,711 tumors from the AACR Project GENIE.

The AACR Project GENIE is a publicly accessible cancer registry that provides real-world clinico-genomic data. The registry (version 13.0-public) includes 111,711 primary or metastatic tumors collected from 16 hospitals or cancer centers (Fig. 1c), representing diverse populations: 55,973 Asians (5.3%); 5,545 Blacks (5.0%); 78,003 Whites (69.8%); 5,311 individuals from other racial groups (4.8%); and 16,879 individuals of unknown race (15.1%). This diversity ensures representative sampling of the cancer patient populations at the participating institutions. The registry encompasses 102 cancer types, grouped into 23 analysis cancer type groups (See Supplementary Note), including 14,983 lung and 12,144 breast tumors (Fig. 1d). It features 757 subtypes of different cancers as defined by OncoTree[22], which is much more comprehensive than subtypes analyzed in previous studies. For instance, while the TCGA ovarian cancer study[23] focuses on high-grade serous ovarian cancer, the AACR Project GENIE dataset involves 34 ovarian cancer subtypes, including high-grade serous ovarian cancer, clear cell ovarian cancer, low-grade serous ovarian cancer, and endometrioid ovarian cancer, among others (Fig. 1e). In summary, the AACR Project GENIE offers a comprehensive and representative dataset for examining targeted sequencing-based mutational signatures across diverse cancer types and subtypes in real-world clinical settings.

**SATS identifies signatures of tumor mutation burden**

One key advancement of SATS lies in its ability to discern signatures of tumor mutation burden (TMB), circumventing the requirement of identical genomic region sequenced across tumor samples for mutational signature analysis. SATS achieves this through approximating $\mathbf{V}$ by $\mathbf{L} \circ \mathbf{WH}$ (i.e., $\mathbf{V} \approx \mathbf{L} \circ \mathbf{WH}$), where $\circ$ denotes element-wise product. SATS allows for the analysis of diverse gene panels, each covering different sequenced genomic regions, by using the panel context matrix $\mathbf{L}$ (measured in megabase (Mb) pairs) to account for these differences. The resulting signature profile matrix $\mathbf{W}$ thus characterizes TMB signatures. In contrast, conventional mutational signature analysis algorithms implicitly require identical sequenced regions across tumor samples (e.g., through WES or WGS). These algorithms employ the canonical NMF method to factorize a mutation type matrix $\mathbf{V}$ into a $96 \times K$ signature profile matrix $\mathbf{W}'$ and a $K \times N$ signature activity matrix $\mathbf{H}'$ (i.e.,$\mathbf{V} \approx \mathbf{W}'\mathbf{H}'$). Thus, the estimated $\mathbf{W}'$ delineates tumor mutation count (TMC) signatures. Since TMB signature profiles account for the variability in mutation context across different panels (Supplementary Fig. 1), whereas TMC signature profiles do not (Methods), TMB signatures are particularly useful for studies involving multiple different targeted gene panels.

We compared the shape of TMB and TMC signature profiles using the Shannon equitability index (Methods). A higher index value corresponds to a flatter signature profile, whereas a lower value indicates a distinct or spikier profile. Although TMB and TMC signature profiles are largely similar (Pearson correlation coefficient r = 0.915, Supplementary Fig. 2a), there are notable exceptions. For example, the TMC SBS5 profile (Shannon equitability index $E_H = 0.941$) is relatively consistent across all 16 trinucleotide contexts of C to T mutations, whereas the TMB SBS5 profile ($E_H = 0.903$) shows increased C to T mutations at the NCG trinucleotides (N represents any nucleotide, Supplementary Fig. 2b), since these trinucleotides are depleted in the human genome due to frequent deamination of 5-methylcytosine to thymine[24,25] (Supplementary Fig. 2c). In addition, TMB signature SBS10b and SBS15 ($E_H = 0.192$ and 0.391 respectively) exhibit more pronounced spikes compared to their TMC counterparts ($E_H = 0.491$ and 0.624 respectively, Supplementary Fig. 2d).

**Factors impacting signature detection and signature burden estimation by SATS**

We investigated factors that may affect the performance of SATS in detecting signatures and estimating signature burdens. These factors include the size of the targeted gene panels, the prevalence of the signatures, the shape of the TMB signature profile (measured by the Shannon equitability index), and the cancer types. We generated pseudo-targeted sequencing data SBSs that were called in The Cancer Genome Atlas (TCGA) WES studies[1,26] and located in the regions covered by various targeted sequencing panels (Methods). In addition, we generated pseudo-targeted sequencing data based on 560 breast tumors[27] with WGS data (Methods and Supplementary Fig. 3a). Our analysis focused on common signatures contributing more than 5% of SBSs based on WES or WGS analyses for a given cancer type. While the sample size could also impact signature detection, we could not assess it using limited pseudo-targeted sequencing data. Thus, we conducted *in silico* simulations to examine the impact of sample size.

First, we analyzed pseudo-targeted sequencing data based on WES and showed that SATS is capable of detecting common signatures, though detection probabilities vary across cancer types, targeted gene panels, and the specific signatures. For instance, larger gene panels generally uncovered more signatures (Fig. 2a). Additionally, there was an inverse relationship (r = -0.452) between the detection probability of a signature and its Shannon equitability index (Fig. 2b). This suggests that "spikier" signatures are more likely to be detected than "flatter" ones, consistent with observations based on WES/WGS data[9]. To simultaneously quantify the impact of these factors on the detection probabilities, we applied a generalized linear mixed model (GLMM) that included cancer type, panel size, signature prevalence, and the Shannon equitability index of the signature (Methods). Our results revealed that cancer types accounted for 53.26% of the variance of detection probabilities at the logit scale (Fig. 2c). This is because certain cancer types possess more distinctive and easily distinguishable signatures. Additionally, cancer types with high TMB (e.g., lung squamous cell carcinoma, median TMB: 10.07 mutations/Mb) had a higher probability of signature detection compared to those with lower TMB (e.g., thyroid adenocarcinoma, median TMB: 0.47 mutations/Mb). Within a specific cancer type, GLMM indicated that spikier (odds ratio (OR) = 0.962, 95% confidence interval (CI) = 0.956-0.967 for a 0.01 increase of the Shannon equitability index, Fig. 2d) and more prevalent (OR = 1.12, 95% CI = 1.14-1.27 for a one percent increase of signature prevalence) signatures are more detectable, especially when using large gene panels (OR = 1.21, 95% CI = 1.14-1.27 for 1Mb increase in gene panel size). This conclusion was also evident in our analysis of pseudo-targeted sequencing data based on WGS (Supplementary Fig. 3b). This finding helps explain the challenge in detecting signatures in thyroid adenocarcinoma, where spikier signatures like SBS1 are less common (the prevalence 6.58%), while the most common signature, SBS5 (28.26%), is flat and may be confused with other flat signatures like SBS3 or SBS40.

Next, we evaluated the signature refitting steps of SATS for estimating signature burdens - the number of mutations attributed to each signature. We compared the signature burdens calculated using WES[1] with these estimated using SATS from pseudo-targeted sequencing data of the same tumors (as an example, see Supplementary Fig. 4a for SBS4 in lung cancer based on the MSK-IMPACT468 panel). A strong correlation between the two would indicate that targeted sequencing panels are a feasible alternative to WES for assessing signature burdens. We found that the median Pearson correlation coefficient was 0.7 for panels with sizes greater than 1Mb (Fig. 2e), with higher correlation coefficients for certain signatures, such as SBS4 in lung adenocarcinoma (r = 0.91) and SBS7a and SBS7b in melanoma (r = 0.98 and 0.95 respectively, Supplementary Fig. 4b and 4c). Similar results were also observed in pseudo-targeted sequencing data based on WGS (Supplementary Fig. 3c).

It is important to note that the sample sizes for pseudo-targeted sequencing data, which consist of hundreds of tumors, are significantly smaller than those for available targeted sequenced tumors, which include thousands of tumors. As a result, the number of mutational signatures detected in pseudo-targeted sequencing data is restricted. To explore the impact of sample size on mutational signature detection, we conducted *in silico* simulations with varying sample sizes, using breast cancer as a case study (consisting of 12 mutational signatures with at least 1% prevalence in the TCGA breast cancer study, Supplementary Fig. 5a). We simulated the mutation burden of 96 SBS mutation types for up to 1 million tumors across 21 different targeted sequencing panels, each with the size larger than 1Mb (Methods). In this way, we could use the "truly" mutational signatures present in *in silico* simulations as benchmarks. Our findings demonstrated that as the number of targeted sequenced tumors increases, SATS can eventually detect almost all common mutational signatures (>5% prevalence) in breast cancer, including the HRD-associated signature

SBS3, while maintaining a low false positive rate (Supplementary Fig. 5b, detailed in the Supplementary Note). This study underscores the importance of sample size in the extraction of mutational signatures from targeted sequenced tumors, as detecting certain signatures might necessitate a larger cohort of samples.

**Evaluation of SATS and other methods by *in silico* simulations**

To assess the performance of SATS in mutational signature detection and burden estimation, we conducted *in silico* simulations, comparing it with other methods. Using signature profiles and distributions of signature activities from the AACR Project GENIE, we simulated mutation type matrices for lung, breast, colorectal, and lymphoid-derived hematologic cancers (Methods).

First, we ran SATS, SigProfilerExtractor[12] and Mix[17] to detect signatures and compared the detected signatures to the 'prespecified signatures' used in the simulations. We observed that SATS could accurately detect most prespecified signatures, except for few flat or rare signatures. For lung and breast cancers, SATS identified all nine prespecified signatures in every replicate (Fig. 3a). In colorectal cancer, SATS detected five out of six prespecified signatures in all replicates, with SBS44 being more elusive. SBS44, as the second flattest signature in colorectal cancer, is challenging to distinguish from the common flat signature SBS5. In lymphoid-derived hematologic cancer, all prespecified signatures were detected. The only false positive signatures in four cancer types were SBS10c and SBS92 in colorectal cancer for one replicate. In contrast, SigProfilerExtractor and Mix failed to detect signatures SBS29 and SBS89 in lung cancer (Fig. 3a). SigProfilerExtractor did not identify SBS44 in colorectal cancer, and Mix missed the majority of signatures in lymphoid-derived hematologic cancer. Besides false negative detections, SigProfilerExtractor incorrectly identified SBS24 in lung cancer and SBS7b in lymphoid-derived hematologic cancer. These results suggest that SATS outperforms SigProfilerExtractor and Mix in signature detection for targeted sequenced tumors, effectively identifying most prespecified signatures with minimal false positives.

Next, we applied SATS, SigProfilerAssignment[14] and Mix[17] to estimate signature burdens, comparing these estimates with the simulated "ground truth". SATS showed high accuracy in estimating burdens for common or spiky signatures, such as SBS2/13 in breast cancer ($r = 0.96$, Fig. 3b), SBS4 in lung cancer ($r = 0.86$) and SBS10a and SBS10b in colorectal cancer ($r = 0.99$ for both). However, the correlation was lower for flatter or rarer signatures, such as SBS89 in breast cancer ($r = 0.55$) and SBS6 in colorectal cancer ($r = 0.74$). The correlations for signature burdens estimated by SigProfilerAssignment and Mix were generally lower than those by SATS, particularly for signatures like SBS6 in colorectal cancer and SBS84/SBS87 in lymphoid-derived hematologic cancer (Fig. 3b). Overall, these results suggest that SATS can more accurately estimate signature burdens for the majority of signatures than other methods.

Finally, we investigated the impact of including irrelevant signatures in signature refitting. Simulating breast cancer targeted sequencing data using signatures SBS1, SBS2/13, and SBS5, we performed signature refitting using 12 signatures (including the three true signatures) from TCGA WES breast cancer study. We found that a considerable proportion of mutations were incorrectly attributed to non-existent signatures in the simulated data (Supplementary Fig. 6). This result emphasizes the importance of choosing an appropriate set of refitted signatures, tailored for targeted sequencing, to enhance the accuracy of signature refitting in targeted sequencing studies.

**Evaluation of SATS and other methods in tumors with both WGS and targeted sequencing**

We further assessed SATS using 72 kidney tumors that had undergone both whole genome sequencing and targeted sequencing[28]. Due to the sample size constraints, we focused on refitting the common signatures to estimate their burdens in the targeted sequencing data using SATS, SigProfilerAssignment[14], Mix[17] and deconstructSigs[13]. The common signatures included SBS1, SBS5, and SBS40, all of which had been identified in kidney tumors from the AACR Project GENIE, as well as through WGS-based mutational signature analyses of 72 kidney tumors[28].

Our analysis showed a consistent correlation between the estimated burdens of flat signatures (SBS5/40) from targeted sequencing and those from WGS across all methods. Specifically, we observed that samples with a higher burden of mutations attributed to flat signatures in targeted sequencing also had a higher burden of these signatures in WGS (Fig. 3c left panel; Wilcoxon P-value = $2.48 \times 10^{-3}$, $2.35 \times 10^{-7}$, $2.35 \times 10^{-7}$, $1.79 \times 10^{-8}$ for SATS, SigProfilerAssignment, Mix and deconstructSigs, respectively). However, for the less frequent signature SBS1, the association of signature burdens was observed only in SATS (Fig. 3c right panel; Wilcoxon P-value = $3.62 \times 10^{-7}$). SigProfilerAssignment and deconstructSigs assigned zero burdens to SBS1 in all samples but one for SigProfilerAssignment, while Mix categorized all samples as one cluster with minimal SBS1 signature burdens. This indicates the limitations of other refitting methods in accurately estimating burdens for less common signatures like SBS1 in targeted sequencing data.

**The pan-cancer repertoire of targeted sequencing-based mutational signatures**

We applied SATS to create a pan-cancer repertoire of SBS signatures based on the targeted sequenced tumors in the AACR Project GENIE. This repertoire can serve as a valuable resource for refitting mutational signatures derived from targeted sequencing, even for a single tumor.

First, we examined the signature prevalence for individual cancer types (Methods). A significant share of the mutations in many cancer types was found to be attributable to flat signatures (SBS3/5/40), with some notable exceptions (Fig. 4a top panel). Specifically, skin cancer or melanoma is primarily characterized by UV-induced signatures (SBS7a/7b), while endometrial cancer is dominated by signatures related to DNA mismatch (SBS6/14/15) and replication repair deficiency (SBS10a/10b). Additionally, APOBEC-induced signatures (SBS2/13) are most frequent in bladder cancer, whereas tobacco- and APOBEC-induced signatures (SBS4/29 and SBS2/13, respectively) dominate in lung cancer.

Next, we evaluated the presence of mutational signatures for individual cancer types (Methods). Our analysis revealed the ubiquitous presence of SBS1, caused by deamination of 5-methylcytosine to thymine, and flat signatures (SBS3, SBS5 and SBS40 combined) across all cancer types (Fig. 4a bottom panel). In contrast, other SBS signatures were cancer type specific. For instance, signatures of endogenous processes such as SBS2/13 (APOBEC cytosine deaminases), SBS6/14/15/44 (mismatch DNA repair deficiency) and SBS10a/b/c (polymerase epsilon (POLE) exonuclease domain mutations) were observed in nine, eight, and nine cancer types, respectively. Signatures associated with environmental exposures, such as SBS4/29

(smoking or tobacco-chewing) and SBS7a/b (UV radiation), were found in lung cancer, head and neck cancer, skin cancer/melanoma or soft tissue cancer, and cancers of unknown primary. We also detected signatures associated with treatment, including SBS11 in glioma, SBS32 in pancreatic cancer, and thiopurine chemotherapy treatment-induced signature SBS87 in lymphoid-derived hematologic cancer[29]. Signature SBS11 is caused by temozolomide, a common chemotherapeutic agent for glioma[30]. Signature SBS32, previously found in skin cancers[31], is caused by azathioprine used to treat autoimmune conditions. Although signature SBS32 has not been previously identified in pancreatic cancers, there are reports which associate azathioprine with acute pancreatitis[32,33], a known risk factor for pancreatic cancer[34,35].

In addition to SBS signatures, we generated a pan-cancer repertoire of DBS mutational signatures for targeted sequenced tumors (Fig. 4b bottom panel). We found seven DBS signatures, which exhibited a low mutation burden (less than one mutation per mega base, Fig. 4b top panel). We observed that the DBS1 signature, associated with UV exposure, is present in head and neck cancer, skin cancer or melanoma, soft tissue cancer, and cancers of unknown primary, consistent with the presence of UV exposure SBS signatures in these cancer types. Furthermore, the DBS2 signature, associated with smoking, was identified in bladder and lung cancers. We also observed DNA repair deficiency-associated signatures DBS3 in non-colorectal bowel cancer.

**Validation of targeted sequencing-based mutational signatures**

To validate the targeted sequencing-derived mutational signature catalogue, we analyzed an additional 13,425 tumors (6,280 lung, 5,056 colorectal, and 2,089 breast tumors) from a newer version (15.1) of the AACR Project GENIE, excluding ones in the original analysis (GENIE version 13.0). Other cancer types had insufficient samples for validation. We observed high replication for established signatures, with SBS1, SBS2/13, SBS4/29, SBS5, SBS40, and SBS89 being consistently detected in lung cancer; SBS1, SBS2/13, SBS5, and SBS40 in breast cancer; and SBS1, SBS5, SBS6, SBS10a, and SBS10b in colorectal cancer (Fig. 5a). Only SBS44 was absent from the GENIE validation set of colorectal cancers. This high replication rate indicates the reliability of the targeted sequencing-based mutational signature catalogue across these three cancer types. The GENIE validation analysis identified additional signatures, SBS94 in lung cancer and SBS10c and SBS15 in colorectal cancer. However, when we analyzed the entire GENIE version 15.1 dataset including ones in the original analysis, SBS94 was no longer detected in lung cancers, suggesting a false positive finding. Conversely, SBS10c, SBS15, and SBS44 were identified in colorectal cancers using more samples. This observation agrees with *in silico* simulations, highlighting the potential for discovering additional signatures while reducing false positive findings with increased sample size.

In another validation study, we analyzed the signatures in an independent cohort of 2,143 lung cancers and 1,206 colorectal cancers from Chinese patients[20]. Sample size of breast cancer (306 tumors) was too small for validation. Similar to the GENIE validation study, most established signatures were validated, including SBS1, SBS2/13, SBS4/29, SBS5, SBS40, and SBS89 in lung cancer, and SBS1, SBS5, SBS6, SBS10a, and SBS10b in colorectal cancer (Fig. 5b). SBS3, a signature highly similar to SBS5, was detected in the Chinese lung cancer validation set, while SBS44 was absent from the Chinese colorectal cancer validation set. These findings not only replicated the most established signatures but also implied the potential generalizability of the targeted sequencing-based mutational signature catalogue across different races and ethnicities.

**Applications of targeted sequencing-based mutational signatures**

We provide here examples of utilizing targeted sequencing-based mutational signatures generated by SATS to address important clinical questions.

Identification of tissue of origin for tumors of unknown primary. The distinct presence and prevalence of targeted sequencing-based mutational signatures across cancer types suggest their potential as indicators of tissue of origin, particularly for tumors of unknown primary. Notably, when examining the clustering of tumors sequenced at the Memorial Sloan Kettering Cancer Center (Fig. 6a, Methods), we observed that most tumors grouped according to their cancer types. For instance, lung tumors formed a distinct cluster, separate from other cancer types. Similarly, tumors with UV signatures (e.g., head and neck cancer, and skin cancer/melanoma) constituted distinct clusters. These clustering patterns were consistently observed in tumors sequenced at the Dana-Farber Cancer Institute (Supplementary Fig. 7). In contrast, tumors of unknown primary clustered alongside lung tumors, tumors with UV signatures, glioma, pancreatic tumors, and others (Fig. 6a), suggesting their potential tissues of origin.

Targeted sequencing-based mutational signatures enriched in early-onset hypermutated colorectal cancer. A previous study showed that early-onset non-hypermutated colorectal cancers exhibited a lower overall TMB than late-onset cases[36]. However, the opposite trend was observed in hypermutated cases. To examine this discrepancy, we analyzed TMBs attributed to specific mutational signatures in non-hypermutated and hypermutated colorectal cancers, adjusting for race, sex, hospital site, tumor status and tumor subtype (Methods). First, we confirmed the previous findings of the inverse association between overall TMB and early-onset status in non-hypermutated and hypermutated colorectal cancers (Fig. 6b and Supplementary Fig. 8). Next, we found that in non-hypermutated colorectal cancers, TMBs attributed to most signatures (SBS1/5/6/10a/10b) were inversely associated with early-onset status (Fig. 6b left panel), leading to an inverse association between the overall TMB and early-onset status (OR = 0.898, 95% CI = 0.875-0.921, Fig. 6b left panel). In contrast, among hypermutated colorectal cancers, the positive association of overall TMB and early-onset status (OR = 1.004, 95% CI = 1.001-1.007, Fig. 6b right panel) was primarily driven by the TMB attributed to the deficient DNA mismatch repair signature (SBS44, OR = 1.015, 95% CI = 1.002-1.027, Fig. 6b right panel) and the signature related to deficient replication repair gene POLE (SBS10a, OR = 1.017, 95% CI = 1.000-1.034) but attenuated by the clock-like signature SBS5 (OR = 0.981, 95% CI = 0.963-0.999). These results reveal distinct mutational processes operating in early-onset hypermutated colorectal cancers.

Targeted sequencing-based mutational signatures associated with immunotherapy response. Using treatment regimens and response records from the AACR Project GENIE, we examined the association between TMB attributed to specific mutational signatures and progression-free survival (PFS) in 470 non-small cell lung cancer patients who received immune checkpoint inhibitors. To account for potential confounding factors, we adjusted our analysis for smoking history, age, race, sex, cancer stage, cancer subtype, and hospital site. We observed a significant association between TMB attributed to SBS1 and poor PFS (HR = 1.31, 95% CI = 1.10-1.56, Fig. 6c left panel and top right panel). In contrast, TMB attributed to the smoking or tobacco-chewing signatures SBS4/29 exhibited a significant association with favorable PFS (HR = 0.94, 95% CI = 0.90-0.98, Fig. 6c left panel and bottom right panel). TMB attributed to other signatures (SBS2/13, SBS89 and SBS5/40) did not show a significant association with PFS. Notably, when considering the overall TMB combining all signatures, only a nominal association with favorable PFS was observed (HR = 0.98, 95% CI = 0.95-1.00, Fig. 6c left panel and Supplementary Fig. 9a). Consistent results were also observed when analyzing overall survival instead of PFS (Supplementary Fig. 9b). These results suggest that signature-specific TMBs, particularly SBS1 and SBS4/29, could be used as more effective biomarkers for predicting the response to immune checkpoint inhibitors in non-small cell lung cancer, compared to the overall TMB.

## Discussion

In this study, we have introduced SATS, a new tool to identify mutational signatures and estimate signature burdens in targeted sequenced tumors. We evaluated SATS using pseudo-targeted sequencing data and found that spiky signature profiles, a high signature prevalence, and large sequencing panels (> 1Mb) increase the accuracy of signature detection and refitting. Moreover, we showed that SATS outperformed other methods through analyzing *in silico* simulated data and samples with both WGS and targeted sequencing. We utilized SATS to analyze 111,711 targeted sequenced tumors in the AACR Project GENIE and developed a pan-cancer catalogue of SBS and DBS signatures, specifically tailored for targeted sequencing tumors. The signatures of lung, breast and colorectal cancers were further validated in additional samples. Using this repertoire, SATS can estimate signature burden even in a single sample, making it a useful tool in the clinic. Finally, we showed examples of using targeted sequencing-based signatures to address clinical questions (e.g., mutational signatures associated immunotherapy response in non-small cell lung cancer).

Our study has made several important contributions to the analysis of mutational signatures in targeted sequenced tumors. First, unique to SATS is the incorporation of panel size in the analysis. In contrast, the other mutation signature analysis tools for targeted sequencing data assume the same sequencing panels across samples. This adaptability ensures SATS's applicability over a wide range of targeted gene panels, enhancing its utility in clinical settings. Second, unlike clustering-based methods[16,37] that aim to detect a specific mutational signature, SATS can identify multiple mutational signatures simultaneously, providing a more comprehensive analysis of the mutational landscape of targeted sequenced tumors. Third, to the best of our knowledge, this study represents the largest and most comprehensive pan-cancer mutational signature analysis for targeted sequenced tumors to date. We have analyzed 23 cancer types and 757 cancer subtypes, including many that were underrepresented or absent in previous studies, and provide a catalogue of mutational signatures derived from a diverse collection of targeted sequenced tumors at various hospitals and cancer centers that could be used as a reference resource to study even a single tumor in the clinic. This catalogue contrasts with repertoires based on WES/WGS that are often oriented more towards research applications.

This study has several limitations that should be considered. First, the data were collected from clinics primarily in the United States and Western Europe as part of the AACR Project GENIE, which may limit the representativeness of our findings for targeted sequenced tumors from other geographic regions. Nevertheless, validation of signatures in a Chinese cohort for lung and colorectal cancers suggests potential generalizability of mutational signatures across races and ethnicities. Second, while the identified repertoire of mutational signatures for targeted sequencing tumors is extensive, it may not encompass the entire spectrum of possible signatures. The current repertoire mainly includes common signatures with spiky profiles, such as signatures related to hypermutation, that are easy to detect with the current sample size per cancer type in the AACR Project GENIE. This selection bias towards easily detectable signatures could mean that less common or subtler signatures might be underrepresented or missed. Additionally, we observed a higher prevalence of hypermutated signatures in our study compared to previous studies using WGS or WES. This discrepancy could be attributed to the nature of targeted sequencing, which may not capture mutations in non-hypermutated tumors as effectively as WGS or WES, potentially leading to an overestimation of the prevalence of hypermutated signatures. Lastly, although the largest examined to date, the current sample sizes for each cancer type within our study are not large enough to effectively differentiate between flat mutational signatures SBS3, SBS5, and SBS40.

To overcome these limitations, it is crucial to increase the number of tumors sequenced by targeted gene panels and to share the resulting data. The decreasing costs and increasing accessibility of targeted sequencing in clinical practices make the expansion of sample sizes a feasible goal. Initiatives like the AACR Project GENIE are already taking steps towards this goal by collecting and sharing more targeted sequencing data and inviting new participants from underrepresented and underserved populations. Our simulation analyses suggest that as the number of targeted sequenced tumors increases, SATS would provide even increased utility as it could detect additional common signatures with very low false discovery rates. This is supported by our analysis of colorectal cancers using a newer version of AACR Project GENIE with more samples. Moreover, with a larger sample pool, SATS has the potential to differentiate between similar mutational signatures, such as SBS3 (associated with HR deficiency) and other flat signatures. This enhanced capability will improve our understanding of the mutational landscape across diverse cancer types and populations.

In summary, we have developed a tool for analyzing mutational signatures in targeted sequenced tumors and created a pan-cancer repertoire of mutational signatures as a resource tailored for targeted sequencing. Our study has highlighted the clinical relevance of these targeted sequencing-based signatures. The SATS R package is publicly available on GitHub. We anticipate that SATS and the repertoire will enhance applications of mutational signature analysis using targeted sequence data in clinical and research settings.

## Methods

### Genomic data of AACR Project GENIE

We retrieved the AACR Project GENIE dataset (version: 13.0-public) from Synapse (https://synapse.org/genie). This dataset includes tumors that were collected as part of routine clinical practice at 16 hospitals or cancer centers in the U.S., Eruope and U.K. and sequenced by targeted sequencing. If multiple tumors were sequenced from a given patient, we randomly selected one for analysis. Additionally, we selected tumors sequenced by gene panels larger than 50Kb. Patients provided their consent, and the study was approved by an institutional review board (IRB). The dataset includes self-reported sex: 58,576 females, 47,694 males, 2 other, and 5,439 of unknown sex.

The tumors were sequenced at CLIA-/ISO-certified labs with high read depth (median: 519X reads, 1st quantile: 307X, 3rd quantile: 808X). Somatic mutations were called at participating centers with various tools, including Mutect2 (ref[38]) and Strelka[39]. Germline variants and artifacts were filtered out using pooled external controls and databases of known germline variants, such as the Genome Aggregation Database (gnomAD)[40]. For more information on the filtering process, please refer to the "AACR GENIE 13.0-public Data Guide" (https://www.aacr.org/wp-content/uploads/2023/03/13.0_data_guide-1.pdf). The dataset includes 1,213,674 single base substitutions (SBS) and 18,519 double base substitutions (DBS). We further removed somatic mutations with a read depth of less than 100 or an alternative allele read count of less than 5. This resulted in 982,095 SBS and 15,149 DBS from 111,711 tumors (Supplementary Table 1) for mutational signature analysis.

It is worth noting that the choice of mutation calling pipelines may impact the signature analysis results. The influence on signature detection is likely to be less pronounced than on signature burden estimation. This is because signature detection is based on aggregated data from a group of patients, which tends to mitigate the variations caused by different mutation calling approaches. We recommend for users of SATS to review mutation calling pipelines used by the contributing institutions of the AACR Project GENIE (described in the AACR GENIE 13.0-public Data Guide) and to follow best practices[41,42] when applying mutation callers and specifying filtering thresholds.

**A Poisson NMF model for signature analysis of tumor mutation burden**

We define a Poisson Non-Negative Matrix Factorization (pNMF) model for SATS. The pNMF model assumes that the SBS count $v_{pn}$ for the $p^{th}$ mutation type in the $n^{th}$ targeted sequenced tumor follows a Poisson distribution with mean $e_{kpn} = \ell_{pn}\sum_{k=1}^{K} w_{pk}h_{kn}$ for $K$ signatures, $p = 1,2,\dots,96, n = 1,2,\dots,N$ and $k = 1,2,\dots,K$. The $v_{pn}$, $\ell_{pn}$, $w_{pk}$ and $h_{kn}$ represent elements of the corresponding matrices **V** (dimension $N$ by 96), **L** (dimension $N$ by 96), **W** (dimension $N$ by $K$) and **H** (dimension $K$ by 96), respectively. This model specification is equivalent to the one used in signeR[11].

The **W** and **H** are the parameters of interest that will be estimated based on the log-likelihood function of the pNMF model:

$$\log\{P(\mathbf{V}|\mathbf{L},\mathbf{W},\mathbf{H})\} = \sum_{n=1}^{N}\sum_{p=1}^{96}\log\left\{e^{-\ell_{pn}\sum_{k=1}^{K} w_{pk}h_{kn}} \times \frac{\left(\ell_{pn}\sum_{k=1}^{K} w_{pk}h_{kn}\right)^{v_{pn}}}{v_{pn}!}\right\}$$
$$= \sum_{n=1}^{N}\sum_{p=1}^{96}\left\{v_{pn}\log\left(\ell_{pn}\sum_{k=1}^{K} w_{pk}h_{kn}\right) - \ell_{pn}\sum_{k=1}^{K} w_{pk}h_{kn} - \log(v_{pn}!)\right\}. \quad (1)$$

When the genomic regions sequenced across all samples are identical, as in WES or WGS, the maximum likelihood estimate based on equation (1) is equivalent to that based on the canonical NMF, which is detailed below. Thus, the proposed pNMF model includes the canonical NMF as a special case.

**pNMF model extends the canonical NMF**

The pNMF model proposed here incorporates the panel context matrix $\mathbf{L}$, extending the canonical NMF, the standard method for identifying mutational signatures in tumors sequenced by WES or WGS. When all samples are sequenced using the same genomic regions (i.e., $\ell_{pn} = \ell_p$), the log-likelihood function in equation (1) is simplified as

$$\log\{P(\mathbf{V}|\mathbf{L},\mathbf{W},\mathbf{H})\} = -D_{KL}(\mathbf{V}|\mathbf{W}',\mathbf{H}) + C,$$

where $C$ is a constant irrelevant to $\mathbf{W}$ and $\mathbf{H}$. It is worth noting that

$$D_{KL}(\mathbf{V}|\mathbf{W}',\mathbf{H}) = \sum_{n=1}^{N}\sum_{p=1}^{96}\left\{v_{pn}\log\left(\frac{v_{pn}}{\sum_{k=1}^{K} w'_{pk}h_{kn}}\right) + \sum_{k=1}^{K} w'_{pk}h_{kn} - v_{pn}\right\} \qquad (2)$$

is equivalent to the objective function of the canonical NMF[43] with $w'_{pk} = \ell_p w_{pk}$.

## Extraction of *de novo* TMB signatures

We utilize signeR[11] to extract *de novo* signatures $\widehat{\mathbf{W}}$ based on the pNMF model. However, because signeR is computationally demanding due to its use of the Markov Chain Monte Carlo (MCMC) method[11], we grouped samples to improve computational efficiency. Our results below reveal that grouping samples does not affect the TMB signatures profile ($w_{pk}$). Specifically, we define $C$ as the sample index set $\{1,2,\dots,N\}$, and $C_m$ as the mutually exclusive set such that $C = \bigcup_{m=1}^{M} C_m$. For $v_{pm}^{\#} = \sum_{n\in C_m} v_{pn}$, the sum of the mutation count for the targeted sequencing tumors with index $n$ belonging to the set $C_m$, we can show that:

$$E\left(v_{pm}^{\#}\right) = \sum_{k=1}^{K}\sum_{n\in C_m} e_{kpn} = \sum_{k=1}^{K} l_{pm}^{\#} w_{pk} h_{km}^{\#},$$

where $l_{pm}^{\#} = \sum_{n\in C_m} \ell_{pn}$ and $h_{km}^{\#} = \frac{\sum_{n\in C_m} \ell_{pn} h_{kn}}{\sum_{n\in C_m} \ell_{pn}}$. Notably, the TMB signature profile $w_{pk}$ remains unchanged. The panel size of combined samples $l_{pm}^{\#}$ is the sum of the panel size of individual samples $\ell_{pn}$ , and signature activity $h_{km}^{\#}$ is the weighted sum of the signature activities of individual samples $h_{kn}$. The mutation count of combined samples $v_{pm}^{\#}$ follows a Poisson distribution, as the sum of independent Poisson counts is still Poisson distributed.

Grouping samples can significantly reduce computation time. For example, when analyzing 10,000 samples using SATS, analysis by grouping 100 tumors can be completed in 28.5 minutes on a laptop with a Gen Intel(R) Core(TM) i7-1165G7 @ 2.80GHz processor and 16 GB of 4267 MHz RAM. In contrast, analyzing the same samples without grouping tumors takes approximately 13 hours.

## Mapping *de novo* TMB signatures to COSMIC reference TMB signatures

Due to the limited number of somatic mutations detected by targeted gene panels, the detected *de novo* TMB signature profiles may be a linear combination of COSMIC reference TMB signature profiles. To address this limitation, we map the *de novo* signature profile matrix $\widehat{\mathbf{W}} = [\widehat{w}_{pk}]$ to reference TMB signatures $\mathbf{W}_0$ (e.g., a $96 \times 76$ COSMIC TMB signature profile matrix for 76 reference SBS TMB signatures), using penalized non-negative least squares[21]:

Resource

$$\min_{\boldsymbol{\beta}} \left\| \widehat{\mathbf{W}} - \mathbf{W}_0 \boldsymbol{\beta} \right\|_2^2 + \lambda \|\boldsymbol{\beta}\|_1 \text{ subject to } \boldsymbol{\beta} > 0 \text{ and } \lambda \geq 0,$$

where $\boldsymbol{\beta}$ is a coefficient vector and the tuning parameter $\lambda$ is selected based on cross-validations. Compared with the non-negative least squares,

$$\min_{\boldsymbol{\beta}} \left\| \widehat{\mathbf{W}} - \mathbf{W}_0 \boldsymbol{\beta} \right\|_2^2 \text{ subject to } \boldsymbol{\beta} > 0,$$

the penalized non-negative least squares allow us to shrink small values of $\boldsymbol{\beta}$ towards zero and select a smaller number of reference signatures with the profile matrix $\mathbf{W}^*$ that have a significant contribution to the *de novo* signature profiles. To reduce the randomness caused by the cross-validation step to select $\lambda$, we repeat this process 100 times, and select only reference TMB signatures with a coefficient $\beta$ greater than 0.1 in more than 80 replicates.

**Estimation of signature activities by an expectation-maximization algorithm**

We propose an expectation-maximization (EM) algorithm to estimate the signature activity matrix $\mathbf{H} = [h_{kn}]$, given the mutation type matrix $\mathbf{V} = [v_{pn}]$, the panel context matrix $\mathbf{L} = [\ell_{pn}]$, and the mapped reference TMB signature profiles $\mathbf{W}^* = [w_{pk}^*]$. The element $v_{pn}$ in $\mathbf{V}$ can be expressed as the sum of independent latent counts $v_{1pn}, v_{2pn}, \cdots, v_{Kpn}$ attributed to $K$ signatures. These latent counts are treated as the missing data, following Poisson distributions with expectations $\ell_{pn} w_{p1}^* h_{1n}, \ell_{pn} w_{p2}^* h_{2n}, \cdots, \ell_{pn} w_{pK}^* h_{Kn}$, respectively. Introducing latent counts allows us to compute the complete data log-likelihood as:

$$\sum_{p=1}^{96} \sum_{n=1}^{N} \sum_{k=1}^{K} \left\{ -\ell_{pn} w_{pk}^* h_{kn} + v_{kpn} \, log\left(\ell_{pn} w_{pk}^* h_{kn}\right) - log\left(v_{kpn}!\right) \right\}.$$

In addition, the conditional distribution of $v_{kpn}$ given $\mathbf{V}$, $\mathbf{L}$, $\mathbf{W}^*$ and $\mathbf{H}^t$ (the $\mathbf{H}$ at the $t$'th iteration of the EM algorithm) follows a multinomial distribution with parameters $v_{pn}$ and $p_k = w_{pk}^* h_{kn}^t / \sum_{j=1}^{K} w_{pj}^* h_{jn}^t$.

In the E-step, we compute $Q(\mathbf{H}|\mathbf{H}^t)$ as the expected complete data log-likelihood:

$$\begin{aligned} Q(\mathbf{H}|\mathbf{H}^t) &= E\left[ \sum_{p=1}^{96} \sum_{n=1}^{N} \sum_{k=1}^{K} \left\{ -\ell_{pn} w_{pk}^* h_{kn} + v_{kpn} \, log\left(\ell_{pn} w_{pk}^* h_{kn}\right) \right\} \middle| \mathbf{V}, \mathbf{L}, \mathbf{W}^*, \mathbf{H}^t \right] \\ &= \sum_{p=1}^{96} \sum_{n=1}^{N} \sum_{k=1}^{K} \left\{ -\ell_{pn} w_{pk}^* h_{kn} + \log\left(\ell_{pn} w_{pk}^* h_{kn}\right) v_{pn} \frac{w_{pk}^* h_{kn}^t}{\sum_{j=1}^{K} w_{pj}^* h_{jn}^t} \right\}. \end{aligned}$$

In the M-step, the maximizer of $Q(\mathbf{H}|\mathbf{H}^t)$ is obtained by setting the derivative with respect to $h_{kn}$ to 0,

$$\frac{\partial}{\partial h_{kn}} Q(\mathbf{H}|\mathbf{H}^t) = -\sum_{p=1}^{96} \ell_{pn} w_{pk}^* + \frac{1}{h_{kn}} \left( \sum_{p=1}^{96} v_{pn} \frac{w_{pk}^* h_{kn}^t}{\sum_{j=1}^{K} w_{pj}^* h_{jn}^t} \right) = 0,$$

and the updated activity value $h_{kn}^{t+1}$ is given by:

$$h_{kn}^{t+1} = h_{kn}^{t} \frac{\sum_{p=1}^{96} v_{pn} \left( \frac{w_{pk}^{*}}{\sum_{j=1}^{K} w_{pj}^{*} h_{jn}^{t}} \right)}{\sum_{p=1}^{96} \ell_{pn} w_{pk}^{*}}.$$

Note that the M-step depends on the current value of activities $h_{jn}^{t}$ for the $n^{th}$ tumor only. Therefore, even though the EM algorithm updates the entire activity matrix $\mathbf{H}$ for all samples simultaneously, it is equivalent to updating the activity of one tumor at a time. In other words, the EM algorithm of SATS for signature refitting estimates signature activities independently of other tumors, enabling signature activities to be estimated accurately for a single tumor or small subset of samples.

To complete the EM algorithm, the E-step and the M-step are iterated until convergence and output the estimated activity matrix $\widehat{\mathbf{H}}$.

**Calculation of signature burdens**

To calculate the expected number of mutations attributed to a signature (referred to as the signature burden) $\boldsymbol{E} = [E_{kn}]$, we use the estimated activity matrix $\widehat{\mathbf{H}} = [\hat{h}_{kn}]$ from the EM algorithm. The signature burden $E_{kn}$ of the $k^{th}$ signature in the $n^{th}$ tumor is then calculated as the sum of the product of the panel size $\ell_{pn}$, the reference signature profile $w_{pk}^{*}$ and the estimated signature activity $\hat{h}_{kn}$ across 96 SBS types as $E_{kn} = \sum_{p=1}^{96} \ell_{pn} w_{pk}^{*} \hat{h}_{kn}$.

**Relationship between signatures of TMB and TMC**

The $D_{KL}(\mathbf{V}|\mathbf{W}', \mathbf{H})$ in equation (2) with $w'_{pk} = \ell_p w_{pk}$ highlights the relationship between signatures of TMB and TMC: TMB signature profile $w_{pk}$ normalizes TMC signature profile $w'_{pk}$, by the number of mutation context $\ell_p$ (i.e., $w_{pk} = w'_{pk}/\ell_p$). This means that we can create a catalogue of TMB signatures based on WGS, dividing the catalogue of TMC signatures (e.g., COSMIC WGS reference TMC signatures[1]) by the number of trinucleotide contexts from which the mutation type could occur in the human reference whole genome.

**Creating a catalogue of reference TMB signatures**

To create a catalogue of reference TMB signatures in Supplementary Table 2, we normalize COSMIC signature profiles of TMC by the size of mutation contexts in the whole genome. This is done by following these steps:

1. Download the COSMIC SBS and DBS signature profiles (version 3.2) from https://cancer.sanger.ac.uk/signatures/
2. For an SBS signature profile, divide the level of each mutation type (e.g., A[C > G]G) by the size of the corresponding mutation context (e.g., ACG for A[C > G]G) that can occur in the whole genome (Supplementary Table 3)
3. Rescale 96 mutation types to sum to one.
4. Similarly, create DBS signature profiles of TMB (Supplementary Table 4) based on COSMIC DBS signature profiles of TMC and the number of genomic contexts (Supplementary Table 5) for which DBS can occur.

**Shannon equitability index of TMB mutational signatures**

We use Shannon equitability index to measure the diversity or "flatness" of a signature profile[44,45]. The index is calculated as

$$Shannon\ equitability\ index\ = -\frac{\sum_{p=1}^{96} w_p log(w_p)}{\log(96)},$$

where $w_p$ is the level of signature profile at the $p$th mutation type and the sum across all mutation types ($i$=1 to 96) is equal to 1.

A higher value of the index indicates a more even distribution of mutation types. The index ranges from 0 to 1, with a value of 1 indicating a completely flat signature profile where all mutation types are represented equally and a value of 0 indicating a signature profile with a single dominant "spike" where a single mutation type has a proportion of 1 and all other mutation types have a proportion of 0. Among SBS TMB signatures with $n = 96$, some profiles are characterized by a few specific mutation types at high levels, referred to as "spikes" (e.g., SBS1 with the C>T substitution at the NCG trinucleotide has a Shannon equitability index of 0.317, and SBS10a with the T[C>A]T substitution has a Shannon equitability index of 0.192). Other signature profiles are more evenly distributed across all mutation types, referred to as "flat" (e.g., SBS3 has a Shannon equitability index of 0.974, SBS5 has a Shannon equitability index of 0.903, and SBS40 has a Shannon equitability index of 0.969).

**Generation of pseudo-targeted sequencing data**

To investigate the impact of various factors on mutational signature detection, we created pseudo-targeted sequencing datasets using the TCGA WES studies[1,26] and the Sanger breast cancer (BRCA) 560 WGS study[27]. We assume that targeted sequencing would identify SBSs that are identified in the WES or WGS studies, as long as SBSs are located within the targeted genomic regions of the panels. This assumption is reasonable since targeted sequencing typically provides much higher sequencing coverage than WES or WGS. The steps to generate the simulated data are outlined below:

1. Download the TCGA WES data (mc3.v0.2.8.PUBLIC) from the Cancer Genome Data Portal (https://gdc.cancer.gov/about-data/publications/mc3-2017) and WGS data of Sanger BRCA560 study (Caveman_560_20Nov14_clean) from ftp://ftp.sanger.ac.uk/pub/cancer/Nik-ZainalEtAl-560BreastGenomes.
2. Download the genomic information file of AACR Project GENIE (https://www.synapse.org/#!Synapse:syn26706790) which specifies the chromosome, start position, and end position of genomic regions for each targeted sequencing panel.
3. For SBS in WES or WGS studies, select those located in the genomic regions of a targeted sequencing panel to create the SBS mutation type matrix as pseudo-targeted sequencing data. We generated 648 pseudo-targeted sequencing datasets, encompassing 18 TCGA WES cancer types and 36 targeted sequencing panels (panel size: 0.05 Mb to 9.95 Mb). Each TCGA WES cancer type has at least 200 samples, ensuring a sufficient sample size for evaluating the signature detection step of SATS. Note that certain WES cases lacked SBS within the genes covered by a targeted sequencing panel. Hence, the number of cases in the pseudo-targeted sequencing data is less than that of TCGA WES studies (Supplementary Table 8). For instance, out of the 208 TCGA sarcoma WES cases, each panel could only detect SBS in a subset of cases (e.g., 169 cases for the

DFCI-ONCOPANEL-3 panel and 172 cases for the MSK-IMPACT505 panel). Similarly, we generated 36 pseudo-targeted sequencing datasets based on 560 breast tumors with WGS data, using 36 targeted gene panels.

**Analysis of pseudo-targeted sequencing data**

We calculate the signature detection probability, which represents the percentage of common signatures detected by 36 targeted sequencing panels within a cancer type. Next, we employ a generalized linear mixed model (GLMM) to analyze the factors that influence the detection probability of TMB mutational signatures across 648 pseudo-targeted sequencing datasets (by 18 TCGA cancer types and 36 targeted sequencing panels). The GLMM incorporates several fixed effects, including the flatness of the signature profile (quantified using the Shannon equitability index), the prevalence of the mutational signature in the TCGA WES study (as a percentage of SBS attributed to the signature), and the panel size (per megabase). To account for any variation in the results due to the different cancer types under investigation, we include cancer type as a random intercept in the model.

**Evaluation of the impact of sample sizes**

We conducted an *in silico* simulation to investigate the effect of sample size on the ability to detect mutational signatures in breast cancer. The simulation was executed using varying sample sizes, ranging from one thousand to up to one million samples, based on the panel context matrix, signatures profile matrix (consisting of 12 mutational signatures with at least 1% prevalence in the TCGA breast cancer study), and signature activity matrix (following the distributions of the signature activity matrix of the TCGA breast cancer study).

1. We run signature refitting on the TCGA breast cancer dataset (accessible at https://www.synapse.org/#!Synapse:syn11726618), using12 known mutational signatures (SBS1, 2, 3, 5, 7a, 10a, 10b, 13, 15, 29, 30, 44 and 58) that have a prevalence greater than 1% (based on https://www.synapse.org/#!Synapse:syn11801497). Specifically, we applied the EM algorithm to estimate the signature activity matrix $\mathbf{H}_B^*$, from the mutation type matrix $\mathbf{V}_B$, the panel context matrix $\mathbf{L}_{WES}$ of the whole exome sequencing, and the pre-defined TMB signature matrix $\mathbf{W}_B^*$.
2. We simulated mutation type matrix $\mathbf{V}_B^{sim}$ for 21 targeted sequencing panels with a panel size larger than 1Mb, using a range of sample sizes from 1000 tumors to 1 million tumors. Specifically, we simulated mutation type matrix $\mathbf{V}_B^{sim}$ from a Poisson distribution with the mean $\mathbf{L}_S \circ \mathbf{W}_B^* \mathbf{H}_B^b$, where $\mathbf{L}_S$ represents the panel size matrix for a given targeted sequencing panel (S), $\mathbf{H}_B^b$ is sampled from the estimated signature activity matrix $\mathbf{H}_B^*$. As the activities of APOBEC signatures SBS2 and SBS13 are highly correlated, their activities were jointly sampled. Finally, we excluded any tumors with zero mutation count.
3. We applied signeR to extract *de novo* signatures $\widehat{\mathbf{W}}_B^S$ from the simulated mutation type matrix $\mathbf{V}_B^{sim}$. Then, we employed penalized non-negative least squares to select the mapped reference TMB signatures, $\mathbf{W}_B^{S*}$. Finally, we estimated signature activities and burdens using the EM algorithm.
4. To evaluate the ability to detect the pre-specified signatures, we analyzed the proportion of 21 panels that were able to rediscover the prespecified 12 mutational signatures using

SATS. We also tracked the probability of detecting false positive signatures that were not used to simulate mutation counts.

**Evaluation of SATS by *in silico* simulations**
We conducted *in silico* simulations to evaluate whether SATS can detect and estimate the prespecified signatures in simulated datasets. When multiple flat signatures were present (e.g., SBS5/40 in lung cancer and SBS3/5/40 in lymphoid-derived hematologic cancer), we combined them into a single flat signature as the sample size of the AACR Project GENIE is insufficient to distinguish between these flat signatures accurately.

1. We first calculated the expectation matrix $\mathbf{E}_c^*$ as $\mathbf{E}_c^* = \mathbf{L}_c \circ \mathbf{W}_c^* \mathbf{H}_c^*$, where $\circ$ denotes element-wise product, $\mathbf{L}_c$ a panel size matrix, $\mathbf{W}_c^*$ a signature profile matrix and $\mathbf{H}_c^*$ a signature activity matrix of a cancer type $(c)$. The matrices $\mathbf{W}_c^*$ and $\mathbf{H}_c^*$ were estimated from the AACR Project GENIE, allowing us to generate simulated data that accurately reflects actual observations.
2. We generated ten replicates of the mutation type matrix $\mathbf{V}_c^{sim}$ for lung cancer, breast cancer, colorectal cancer, and lymphoid-derived hematologic cancer respectively by simulating data from the Poisson distribution using expectation matrix $\mathbf{E}_c^*$. The number of simulated samples is the same as in the corresponding AACR Project GENIE studies.
3. We applied signeR and penalized non-negative least squares to estimate TMB signatures $\mathbf{W}_c^{est}$ for each simulated mutation type matrix $\mathbf{V}_c^{sim}$. We then compared these estimated signatures with the ground truth signatures $\mathbf{W}_c^*$.
4. Using the simulated mutation type matrices ($\mathbf{V}_c^{sim}$), panel size matrices ($\mathbf{L}_c$), and estimated TMB signatures ($\mathbf{W}_c^{est}$), we estimated the signature activity matrix ($\boldsymbol{H}_c^{est}$) for all tumors using the EM algorithm. We then calculated the signature burden based on $\boldsymbol{H}_c^{est}$, which is compared with the simulated signature burden as the ground truth. For lung cancer, we also estimated $\boldsymbol{H}_c^{est}$ for a subset of samples or even for one sample, as detailed in the Supplementary Note.

**Applications of other methods for *in silico* simulations**
We applied SigProfilerExtractor[12] on the simulated mutation type matrix $\mathbf{V}_c^{sim}$ to extract signatures. Considering the computational intensity of SigProfilerExtractor, we grouped the simulated samples in $\mathbf{V}_c^{sim}$. To obtain the mapped COSMIC signatures (version 3.2), we ran SigProfilerExtractor with its default options, setting the number of signatures to be extracted within the range of 1 to 10. After extracting these signatures, we utilized SigProfilerAssignment[14] to compute the corresponding signature burdens based on the mapped COSMIC signatures.

Moreover, we applied Mix[17] to the same simulated data. Mix clustered samples while simultaneously learning the mixture model; hence we used the original simulated matrix $\mathbf{V}_c^{sim}$ as the input. We specified the number of clusters and signatures ranges from 1 to 20 and 1 to 10, respectively. Bayesian Information Criterions (BICs) were computed to identify the optimal combination of the numbers of clusters and signatures. Under the selected optimal combination, the exposure matrix was calculated. As Mix lacks a mapping step to COSMIC signatures, we annotated Mix signatures as COSMIC signatures with the highest cosine similarity. To obtain the signature burden, the total number of mutations was multiplied by the exposure matrix.

**Validation of SATS in tumors with both WGS and targeted sequencing**
We analyzed 72 kidney tumors with both WGS and targeted sequencing. For WGS, genomic DNA was extracted from fresh frozen tissue using the QIAmp DNA mini kit (Qiagen) and subsequently sequenced on the Illumina HiSeqX platform. The mean sequencing depth was 65.7x for tumor tissue and 40.1x for normal tissue. For targeted sequencing, genomic DNA was purified using Agencourt AMPure XP Reagent (Beckman Coulter Inc., Brea, CA, USA). A targeted driver gene panel (size 1.90 Mb) was designed, encompassing 254 candidate cancer driver genes[46]. The targeted sequences were captured by NimbleGen's SeqCap EZ Choice (custom design; Roche NimbleGen, Inc., Madison, WI, USA). Subsequent targeted sequencing was conducted on an Illumina HiSeq 4000, achieving a mean depth of 500x for both tumor and normal tissue. The details of whole-genome and targeted sequencing and sequencing data preprocessing, alignment and somatic mutation calling were described previously[28].

We utilized SATS, SigProfilerAssignment[14], Mix[17] and DeconstructSigs[13] to refit the common signatures, namely SBS1, SBS5, and SBS40, estimating their burdens in the targeted sequencing data. Next, we calculated signature burdens for SBS1 and the combined flat signatures (SBS5/40) in the targeted sequencing data and compared these with the corresponding signature burdens derived from WGS. This comparison allowed us to assess the consistency of estimating mutational signature burdens between the two sequencing methods.

**Generation and validation of targeted sequencing-based mutational signature repertoire**
We generated a pan-cancer repertoire of targeted sequencing-based mutational signatures using data from the AACR Project GENIE (version 13.0). Mutational signatures were analyzed using the SATS pipeline for one cancer type at a time by grouping 100 tumors to expedite computation. The GENIE validation data (version 15.1) were obtained from Synapse (https://www.synapse.org/Synapse:syn55234548). For validation, we selected samples not included in GENIE version 13.0 for three cancer types with large sample size (lung, colorectal, breast cancers). The Chinese validation data of lung and colorectal cancers were acquired from cBioPortal (https://www.cbioportal.org/study/summary?id=pan_origimed_2020). The same pipeline was applied to the GENIE and Chinese validation datasets, grouping 50 tumors and replicating 10 times with different random seeds due to the smaller size of these datasets. For the Chinese validation dataset, we excluded samples with extremely high tumor mutation burdens (three lung cancers with more than 42 mutations/Mb and twelve colorectal cancers with more than 130 mutations/Mb).

**Visualization of SBS mutational signature profiles using UMAP**
We applied UMAP[47] (Uniform Manifold Approximation and Projection) to reduce the dimensionality of SBS mutational signature profiles and visualized tumors in a two-dimensional scatterplot. To facilitate visualization, tumors within each cancer type are first clustered based on their mutational signature burden profiles using hierarchical clustering with Ward's minimum variance method. A cut-off value of 0.05 is applied for clustering. The UMAP projections are computed based on the median signature burden for each cluster of tumors, with each dot representing a group of tumors with similar signature burden profiles.

**Association between mutational signatures and the risk of early-onset colorectal cancer**

Resource

To investigate the relationship between mutational signatures and the risk of early-onset colorectal cancer (sequencing age < 50 years), we analyzed the mutational signature profiles of 9,562 colorectal cancer patients along with their clinical data. We stratified the patients into non-hypermutated cases (TMB < 10 mutations/Mb, early-onset: 2,495 cases; late-onset: 6,009 cases) and hypermutated cases (early-onset: 790 cases; late-onset: 268 cases).

We employed a generalized linear mixed model (GLMM) to compare early-onset vs late-onset cases for the non-hypermutated and hypermutated colorectal cancer, respectively. The GLMM incorporated tumor status (primary vs metastasis), race, sex, and individual signatures' TMBs (SBS1, SBS6, SBS10a, SBS10b, SBS44, and flat SBS) as fixed effects, while center and subtype were considered random effects. The GLMMs were fitted using the 'lme4' package in R.

**Analysis of mutational signatures as immunotherapy predictive biomarkers**
The AACR Project GENIE Biopharma Collaborative released comprehensive clinical data for 1,846 non-small cell lung cancer (NSCLC) patients (v2.0-public dataset) from four hospitals (MSKCC, DFCI, VICC and UHN). Within this dataset, we focused on a subset of 470 NSCLC patients who underwent treatment with immune checkpoint inhibitor (ICI), including Pembrolizumab, Nivolumab, Atezolizumab, and Durvalumab. We aimed to investigate the predictive value of mutational signature-specific TMB for ICI immunotherapy.

We employed a mixed-effect Cox model to analyze progression-free survival, which measures the time interval between the initiation of ICI treatment and the radiologist or oncologist's assessment of cancer progression or patient death.  In the mixed-effect Cox model, we included variables including age, sex, smoking history, tumor stage (IV vs. others), and individual mutational signature-specific TMBs (SBS1, SBS2/13, SBS4/29, SBS89, and flat SBS) as fixed effects. Additionally, we considered the hospital site and tumor subtype as random effects. We also applied the same model to evaluate overall survival, which measures the time interval between the start of ICI treatment and patient death or the last follow-up date.

**Software**

The R package SATS is publicly available at https://github.com/binzhulab/SATS.

**Author contribution:** SJC, JS, MTL, and BZ contributed to the study concept and design and obtained the funding. DL, MH, DW, LS, TZ, XH and BZ conducted bioinformatic and biostatistical analyses. KY, XRY, SJC, JS, MTL, and BZ performed data interpretation. DL, MH and BZ drafted the initial manuscript. All authors contributed to the critical revision of the manuscript and approved the final manuscript.

**Acknowledgment**
This research was conducted with support from Intramural Research Program of the National Institutes of Health, National Cancer Institute, Division of Cancer Epidemiology and Genetics (DCEG). Additionally, Dr. Lee was supported by the National Research Foundation of Korea (NRF) grant funded by the Korea government (MSIT, No. RS-2023-00213625). The authors would like to acknowledge the American Association for Cancer Research and its financial and material support in the development of the AACR Project GENIE registry, as well as members of the consortium for their commitment to data sharing. Interpretations are the responsibility of study authors. This study utilized the high-performance computational capabilities of the Biowulf Linux cluster at the National Institutes of Health, Bethesda, MD: https://biowulf.nih.gov. We would like to thank Bill Wheeler (Information Management Services) for computation support.

**Figure legends:**

**Fig. 1 Overview of SATS and the AACR Project GENIE study. a.** The schematic workflow of SATS starts with summarizing somatic mutations (e.g., single base substitutions) identified through targeted sequencing into a mutation type matrix **V**. In addition, SATS requires a panel context matrix **L** that specifies the number of trinucleotide contexts for individual panels. SATS is based on a Poisson Nonnegative-Matrix Factorization (pNMF) model, which approximates the matrix **V** by $\mathbf{L} \circ \mathbf{W} \times \mathbf{H}$ (i.e., $\mathbf{V} \approx \mathbf{L} \circ \mathbf{W} \times \mathbf{H}$), where $\circ$ denotes the element-wise product and $\times$ represents the matrix multiplication operator. **b.** The analysis procedure of SATS involves signature detection for a patient cohort and signature refitting for individual patients. In this illustrative example, SATS initially identifies two *de novo* tumor mutation burden (TMB) signatures in the cohort, subsequently mapping them to reference TMB signatures 1, 2/13 and 5. Next, SATS carries out signature refitting for six patients (e.g., Pt.1, Pt.2, …, Pt.6), estimating the activities of the mapped reference TMB signatures and the expected number of mutations attributed to each signature, termed the signature burden. For instance, the activities of SBS1, SBS2/13 and SBS5 for patient 3 (Pt.3) are 0.27, 0.84 and 0.18, respectively. Additionally, we estimate 0.67, 1.16 and 3.17 SBS attributed to signature SBS1, SBS2/13 and SBS5, respectively. **c.** The participating hospitals or cancer centers were located in the United States, United Kingdom, and Europe. The sample size for each site is included in parentheses. The sites include: Children's Hospital of Philadelphia (CHOP), The Herbert Irving Comprehensive Cancer Center at Columbia University (COLU), Cancer Research UK Cambridge Centre (CRUK), Dana-Farber Cancer Institute (DFCI), Duke Cancer Institute (DUKE), Institute Gustave Roussy (GRCC), Johns Hopkins Sidney Kimmel Comprehensive Cancer Center (JHU), The University of Texas MD Anderson Cancer Center (MDA), Memorial Sloan Kettering Cancer Center (MSK), Netherlands Cancer Institute (NKI), Swedish Cancer Institute (SCI), University of Chicago Comprehensive Cancer Center (UCHI), University of California, San Francisco (UCSF), Princess Margaret Cancer Centre, University Health Network (UHN), Vall d' Hebron Institute of Oncology (VHIO), Vanderbilt-Ingram Cancer Center (VICC), Wake Forest Baptist Medical Center (WAKE), and Yale Cancer Center (YALE). **d.** The histograms show the number of tumors included in the mutational signature analyses for each cancer type. The colors correspond to primary tumors, metastatic tumors, and unspecified or hematological malignancies. **e.** Subtypes of lung cancers, breast cancers, and gynecological cancer-ovarian cancers included in the AACR Project GENIE study. The subtypes were reported by each clinical institution based on OncoTree and are not necessarily mutually exclusive. For example, NSCLC includes both LUAD, LUSC and other subtypes. The size of each tile is proportional to the sample size of the corresponding subtype. The abbreviations for lung cancer, ALUCA: Atypical Lung Carcinoid, CSCLC: Combined Small Cell Lung Carcinoma, LCLC: Large Cell Lung Carcinoma, LNET: Lung Neuroendocrine Tumor, LUAD: Lung Adenocarcinoma, LUAS: Lung Adenosquamous Carcinoma, LUCA: Lung Carcinoid, LUNE: Large Cell Neuroendocrine Carcinoma, LUPC: Pleomorphic Carcinoma of the Lung, LUSC: Lung Squamous Cell Carcinoma, NSCLC: Non-Small Cell Lung Cancer, NSCLCPD: Poorly Differentiated Non-

Small Cell Lung Cancer, PPB: Pleuropulmonary Blastoma, SARCL: Sarcomatoid Carcinoma of the Lung, SCLC: Small Cell Lung Cancer. The abbreviations for breast cancer, BA: Breast Angiosarcoma, BRCA: Invasive Breast Carcinoma, BRCANOS: Breast Invasive Cancer, NOS, BRCNOS: Breast Invasive Carcinoma, NOS, BREAST: Breast, DCIS: Breast Ductal Carcinoma In Situ, IBC: Inflammatory Breast Cancer, IDC: Breast Invasive Ductal Carcinoma, ILC: Breast Invasive Lobular Carcinoma, IMMC: Breast Invasive Mixed Mucinous Carcinoma, LCIS: Breast Lobular Carcinoma In Situ, MBC: Metaplastic Breast Cancer, MDLC: Breast Mixed Ductal and Lobular Carcinoma, MPT: Malignant Phyllodes Tumor of the Breast, PT: Phyllodes Tumor of the Breast, SPC: Solid Papillary Carcinoma of the Breast. The abbreviations for ovarian cancer, CCOV: Clear Cell Ovarian Cancer, EOV: Endometrioid Ovarian Cancer, GRCT: Granulosa Cell Tumor, HGSFT: High-Grade Serous Fallopian Tube Cancer, HGSOC: High-Grade Serous Ovarian Cancer, LGSOC: Low-Grade Serous Ovarian Cancer, MBOV: Mucinous Borderline Ovarian Tumor, MOV: Mucinous Ovarian Cancer, MXOV: Mixed Ovarian Carcinoma, OCS: Ovarian Carcinosarcoma/Malignant Mixed Mesodermal Tumor, ODYS: Dysgerminoma, OGCT: Ovarian Germ Cell Tumor, OIMT: Immature Teratoma, OMT: Mature Teratoma, OOVC: Ovarian Cancer, Other, OSMCA: Ovarian Seromucinous Carcinoma, OVT: Ovarian Epithelial Tumor, OYST: Yolk Sac Tumor, SBMOV: Serous Borderline Ovarian Tumor, Micropapillary, SBOV: Serous Borderline Ovarian Tumor, SCCO: Small Cell Carcinoma of the Ovary, SCST: Sex Cord Stromal Tumor, SCT: Steroid Cell Tumor, NOS, SLCT: Sertoli-Leydig Cell Tumor, SOC: Serous Ovarian Cancer.

**Fig. 2: Assessing the determinants of SATS performance in signature detection and signature burden estimation a.** Detection probability of common signatures, which contribute at least 5% of single base substitutions (SBSs) per a cancer type in the TCGA WES study (e.g., SBS1, SBS5, SBS10a, SBS10b, SBS15 and SBS40 in glioblastoma). Detection probability is defined as the percentage of these signatures detected by SATS in pseudo-targeted sequencing samples for each cancer type across 36 panels. For instance, if SATS detects three out of six common signatures in glioblastoma (e.g., SBS1, SBS10b, SBS15), the detection probability is 50%. The median sample size for pseudo-panel data across all panels is denoted in parentheses on the y-axis, and the size of genomic regions covered by each sequencing panel is indicated on the x-axis. **b.** The percentage of common TCGA WES signatures detected by 36 targeted sequencing panels *versus* the Shannon equitability index of the signature profile. The blue line refers to the linear regression line. The color dots refer to the detected signatures and gray dots refer to the undetected signatures. **c.** The proportion of variance in detection probabilities of common TCGA WES signatures that can be explained by determinant factors, including the Shannon equitability index of the signature profile (flatness), the frequency of TCGA WES signatures (prevalence), panel size, and cancer type. **d.** The odds ratio of determinant factors. Bars represent the 95% confidence intervals (CIs). **e.** The median Pearson correlation coefficient for all detected common WES signatures across 18 TCGA cancer types was compared to panel size. The Pearson correlation coefficient is a measure of the correlation between the number of single base substitutions (SBS) attributed to a signature, estimated from the pseudo-panel data, and the number of SBS attributed to the same signature previously reported in the TCGA WES study. The blue curve represents LOWESS (Locally Weighted Scatterplot Smoothing) curve, and the shaded area is 95% CIs. CA: cervical cancer; HCC: hepatocellular carcinoma; RCC: renal cell carcinoma; CNS: central nervous system; GBM: glioblastoma; AdenoCa: adenocarcinoma;

SCC: squamous cell carcinoma; Thy: thyroid; Prost: prostate; Colorect: colon or rectum; DLBC: diffuse large B cell lymphoma.

**Fig. 3 Comparison of SATS and other methods. a.** Detection frequency of mutational signatures in 10 replicates for lung, breast, colorectal, and lymphoid-derived hematologic cancers. The signatures on the x-axis are used to simulate mutation counts and are considered as the "ground truth". The dot size is proportional to the detection frequency by SATS, SigProfilerExtractor and Mix. **b.** Pearson correlation coefficients between the simulated SBS signature burdens (as the benchmark) and the burdens estimated by SATS, SigProfilerAssignment and Mix. Bars represent the average the Pearson correlation coefficient, and the intervals are the average plus or minus one standard deviation. **c.** Boxplots illustrating mutational signature burdens of flat signatures (SBS5/SBS40) and SBS1 obtained from WGS, separated by signature burden group based on targeted sequencing (flat signature high: flat signature burden > 4 mutations per sample; SBS1 high: SBS1 burden > 1 mutation per sample). The median of the burdens is marked by the line in each box, which spans from the first to the third quartiles. Whiskers extend to the furthest points within 1.5 times the interquartile range from the quartiles. Each dot represents a data point of mutational signature burdens in WGS.

**Fig. 4 Repertoire of mutational signatures in the AACR Project GENIE. a.** Single base substitution (SBS) signatures. The top bar chart displays the stacked tumor mutation burden (TMB) attributed to specific signatures, with the colors indicating different mutational signatures. The bottom panel illustrates the presence of SBS signatures for individual cancer types, with dot sizes representing the proportion of tumors in which an SBS signature is present. The sample size of targeted sequenced tumors is indicated between the top and bottom panels, and the proposed etiology of the mutational signature is included in parentheses. **b.** Double base substitution (DBS) signatures. The top stacked bar chart shows the TMB of DBS signatures, and the bottom panel shows the proportion of tumors for which a DBS signature is present. 5meC: 5-Methylcytosine; APOBEC: apolipoprotein B mRNA-editing enzyme, catalytic polypeptide, MMR: mismatch repair; UV: ultraviolet radiation; POLE-exo*: mutations in polymerase epsilon exonuclease domain; TMZ: temozolomide; BER: base excision repair; AZA: azathioprine; AID: activation-induced deaminase; TP: thiopurine.

**Fig. 5 Validation of targeted sequencing-based mutational signatures using the GENIE version 15.1 validation dataset (a) and the Chinese validation dataset (b).** The signatures identified in GENIE version 13.0 discovery dataset are listed on the left of the circos plots, while signatures identified in the validation datasets are listed on the right. If a signature was validated, a line is drawn between the signature names. The width of the lines is proportional to the number of replicates in which the signature was validated.

**Fig. 6 The associations between targeted sequencing-based mutational signatures and clinical outcomes. a.** UMAP visualization of SBS mutational signature profiles**.** The UMAP (Uniform Manifold Approximation and Projection) scatterplot displays 2-dimensional projections of the signature burden profiles for tumors obtained from Memorial Sloan Kettering Cancer Center. Each color represents a cancer type. **b.** The odds ratios (ORs) for the association between the status of colorectal cancer onset (early-onset vs. late-onset) with overall tumor mutation burden (TMB) or burdens attributed to individual signature (e.g., SBS1, SBS6,

SBS10a, SBS10b, SBS44, SBS5). The left panel illustrates the results for non-hypermutated colorectal cancer, while the right panel displays the findings for hypermutated colorectal cancer. The bars represent 95% confidence intervals (CIs) for the corresponding OR estimates. **c.** The left panel shows the hazard ratio (HR) of overall tumor mutation burden (TMB) or burdens attributed to individual signature (e.g., SBS1, SBS2/13, SBS4/29, SBS89 and SBS5/40) with 95% CIs for progression-free survival (PFS) in lung cancer patients treated with immune checkpoint inhibitors (ICI). The remaining two panels show the proportion of lung cancer patients who remain progression free after receiving ICI, stratified by high or low mutational signature-specific TMB levels. The top right panel represents TMB attributed to signature SBS1, while the bottom right panel represents TMB attributed to smoking or tobacco-chewing signatures SBS4/29. The P-value was calculated using the log-rank test.

**Supplementary figure legends:**

**Supplementary Fig. 1 Boxplots of mutation context ratios between targeted sequences vs. whole genome sequence.** Each dot represents the ratio between the proportion of a mutation context (e.g., CCG) in the genomic regions targeted by a sequencing panel and the proportion of the same mutation context in the whole genome.

**Supplementary Fig. 2 Mutation profiles and contexts in the human whole genome. a.** Scatterplot of the Shannon equitability index of TMC and TMB signature profiles. The black line represents the diagonal line, the blue line the linear regression line, and the shaded area is 95% confidence intervals. The dots are annotated when the differences of Shannon equitability index between TMC and TMB signature profiles are more than 0.1. **b.** Profiles of SBS5 signature based on tumor mutation count (TMC) and tumor mutation burden (TMB), respectively, with 96 mutation types on the x-axis and contributions on the y-axis. **c.** 32 mutation contexts for single base substitutions (SBS) in the human whole genome, with significantly depleted ACG, TCG, GCG, and CCG trinucleotides. **d.** Profiles of SBS10b and SBS15 signatures based on TMC and TMB, respectively.

**Supplementary Fig. 3 Results of pseudo-targeted sequencing data based on Sanger breast cancer 560 WGS study. a.** The Shannon equitability index of the signature profile and the percentage of single base substitutions (SBS) attributed by a signature for the Sanger breast cancer (BRCA) 560 WGS study. The signatures with > 5% prevalence are highlighted in color, while others are shown in gray. **b.** The odds ratio of the Shannon equitability index of the signature profile, frequency of Sanger BRCA 560 WGS study signatures, and panel size. The bars represent 95% confidence intervals (CIs). **c.** The median of the Pearson correlation coefficient for all common WGS signatures detected. The blue curve represents LOWESS (Locally Weighted Scatterplot Smoothing) curve with the shaded area for 95% CIs.

**Supplementary Fig. 4 Results of pseudo-targeted sequencing data based on TCGA WES study. a.** Scatterplot of the number of mutations attributed to the smoking-related signature SBS4 (on the y-axis) in the MSK-IMPACT 468 panel compared to the number of mutations attributed to the same signature in the TCGA WES study (on the x-axis). The black line represents the ratio of the MSK-IMPACT 468 panel size to the WES size. The blue line represents the linear regression line, and the shaded area represents 95% confidence intervals (CIs). **b.** and **c.** Medians of Pearson correlation coefficients for signatures SBS4 (**b**) and SBS7a/b

(**c**) are shown. Pearson correlation coefficient measures the correlation between the number of mutations attributed to a signature using the pseudo-targeted sequencing data and the number of mutations attributed to the same signature reported previously in the TCGA WES study. The curve represents LOWESS (Locally Weighted Scatterplot Smoothing) curve, with the shaded area representing 95% CIs.

**Supplementary Fig. 5 Impact of sample sizes on mutational signature detection. a.** The scatterplot of the flatness (measured by Shannon equitability index) of signature profiles and percentage of single base substitutions (SBS) attributed to the signatures in the TCGA breast cancer (BRCA) WES study. The reference line with Shannon equitability index one refers to the theoretical maxima (by a completely flat signature). **b.** The probability of signature detection. Each dot represents the probability of signature detection, measuring the proportion of 21 targeted sequencing panels (with panel size larger than 1Mb) that can identify the signature at a given sample size. The blue line is the LOWESS (Locally Weighted Scatterplot Smoothing) curve, and the shaded area represents 95% confidence intervals (CIs).

**Supplementary Fig. 6 The proportion of tumors in which the mutation signatures were detected in simulated breast cancer data.** Mapped signatures SBS1, SBS2/13, and SBS5 were used for simulation. Mapped signatures or WES-based signatures were used for signature refitting, respectively. WES-based signatures include SBS1, SBS2/13, SBS5, SBS3, SBS7a, SBS10a, SBS10b, SBS15, SBS29, SBS30, SBS44 and SBS58, which are present in more than 1% of mutations in TCGA WES breast cancer study. The horizontal lines represent the actual proportions in the simulated data. The error bars in the figure represent the mean plus or minus one standard deviation.

**Supplementary Fig. 7 UMAP visualization of SBS mutational signature profiles.** The UMAP (Uniform Manifold Approximation and Projection) scatterplot displays 2-dimensional projections of the signature burden profiles for tumors obtained from the Dana-Farber Cancer Institute. The colors represent cancer types.

**Supplementary Fig. 8 The opposite association between overall tumor mutation burden (TMB) and early-onset (age < 50) colorectal cancer in non-hypermutated and hypermutated tumors.** In non-hypermutated tumors, late-onset cases exhibit a higher overall TMB, while in hypermutated tumors, early-onset cases demonstrate a higher overall TMB.

**Supplementary Fig. 9 The associations between targeted sequencing-based mutational signatures and immunotherapy response. a.** The proportion of lung cancer patients who remain progression free after receiving immune checkpoint inhibitors (ICI), stratified by high (> 10 Mut/Mb) or low overall TMB levels. The P-value was calculated using the log-rank test. **b.** The hazard ratio (HR) of TMB attributed to individual signatures for overall survival (PFS) in lung cancer patients treated with ICI.

**Supplementary Fig. 10 Detection probability of TCGA breast cancer signatures in simulations.** The probability of signature detection is shown for increasing sample sizes (**a.** up to 10,000 tumors, **b.** up to 1,000,000 tumors). Each dot represents the proportion of targeted sequencing panels (with a panel size larger than 1Mb) that are able to identify the signature at the corresponding sample size. Blue curves are LOWESS (Locally Weighted Scatterplot Smoothing) curves, and shaded areas represent 95% confidence intervals.

**Supplementary Fig. 11 The Pearson correlation coefficient between simulated and estimated SBS signature expectancies in lung cancer with various sample sizes for signature refitting**. The bars in the figure represent the mean Pearson correlation coefficient for simulation replicates, while the x-axis indicates the number of samples used for signature refitting, including 100 samples, 10 samples, or even one sample at a time. The error bars in the figure represent the mean plus or minus one standard deviation.

Fig.1

a
Input
Targeted Sequencing
Mutation type matrix V
Panel context matrix L
Mutation types
Patients
Model
Poisson Nonnegative-Matrix Factorization (pNMF) model
Mutation type matrix V
Panel context matrix L
Signature profile matrix W
Signature activity matrix H
Signature 1
Signature 2
Signature K
Signatures
Patient 1
Patient 2
Patient N
b
Analysis procedure and output
Signature detection for a patient cohort
Signature refitting for individual patients
a. De novo TMB signature discovery, adjusting panel sizes
b. Mapping to the reference TMB signatures
c. Estimating activities of reference TMB signatures
d. Calculating burdens of reference TMB signatures
C>A C>G C>T T>A T>C T>G
De novo sig.1
De novo sig.2
Contributions
SBS1
SBS2/13
SBS5
De novo TMB signature profiles
Reference TMB signature profiles
Signature activity matrix H
Signature burden matrix E
Pt.1 Pt.2 Pt.3 Pt.4 Pt.5 Pt.6
c
SCI (645)
PROV (7348)
UCSF (7375)
UCHI (269)
UHN (2721)
DFCI (28957)
YALE (757)
MSK (52334)
CHOP (522)
JHU (1116)
VICC (4078)
DUKE (2548)
CRUK (2097)
NKI (23)
GRCC (901)
VHIO (20)
d
Number of samples
Metastasis
Primary
Unspecified or Heme
Lung Cancer
Breast Cancer
Bowel Cancer–Colorectal
CNS or Brain Cancer–Glioma
Pancreatic Cancer
Hematologic Cancer–Myeloid
Gynecologic Cancer–Ovarian
Skin Cancer or Melanoma
Hematologic Cancer–Lymphoid
Gynecologic Cancer–Endometrial
Prostate Cancer
Cancer of Unknown Primary
Soft Tissue Cancer
Esophagogastric Cancer
Bladder Cancer
Rare Cancer
Hepatobiliary Cancer
Head and Neck Cancer
Kidney Cancer
CNS or Brain Cancer–Other
Thyroid Cancer
Bowel Cancer–Other
Gynecologic Cancer–Other
e
Lung Cancer (14983)
LUAD
LUSC
NSCLC
SCLC
NSCLCPD
LUNE
LUCA
LNET
Breast Cancer (12144)
IDC
BRCA
ILC
MDLC
BRCNOS
BRCANOS
MBC
Gynecologic Cancer–Ovarian (5061)
HGSOC
CCOV
LGSOC
EOV
SOC
HGSFT
OCS
GRCT
MOV
OVT
MXOV
OOVC
SBOV
SLCT
MBOV

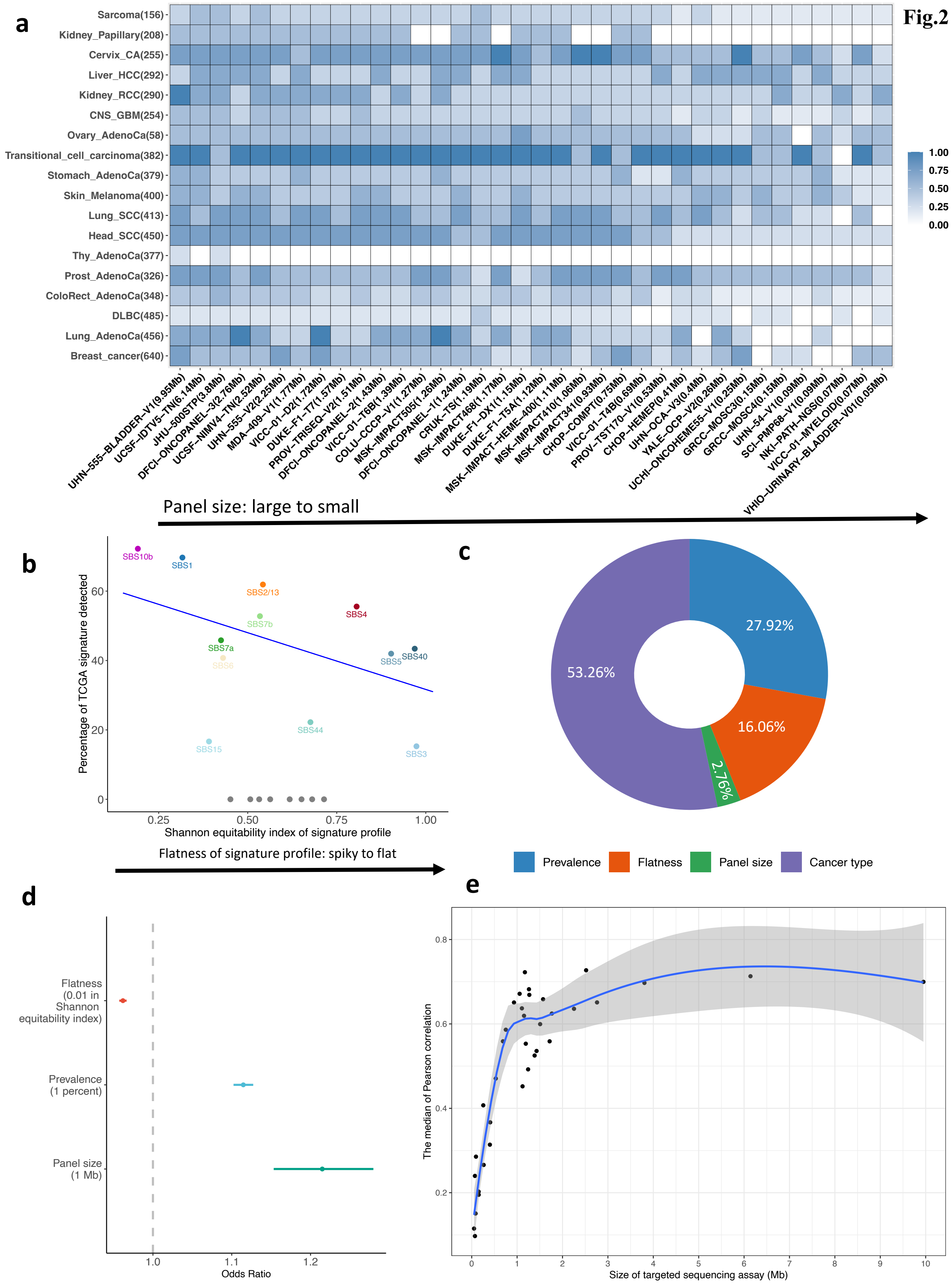

Fig.2
a
Sarcoma(156)
Kidney_Papillary(208)
Cervix_CA(255)
Liver_HCC(292)
Kidney_RCC(290)
CNS_GBM(254)
Ovary_AdenoCa(58)
Transitional_cell_carcinoma(382)
Stomach_AdenoCa(379)
Skin_Melanoma(400)
Lung_SCC(413)
Head_SCC(450)
Thy_AdenoCa(377)
Prost_AdenoCa(326)
ColoRect_AdenoCa(348)
DLBC(485)
Lung_AdenoCa(456)
Breast_cancer(640)
1.00
0.75
0.50
0.25
0.00
Panel size: large to small
b
Percentage of TCGA signature detected
Shannon equitability index of signature profile
Flatness of signature profile: spiky to flat
c
27.92%
16.06%
2.76%
53.26%
Prevalence
Flatness
Panel size
Cancer type
d
Flatness (0.01 in Shannon equitability index)
Prevalence (1 percent)
Panel size (1 Mb)
Odds Ratio
e
The median of Pearson correlation
Size of targeted sequencing assay (Mb)

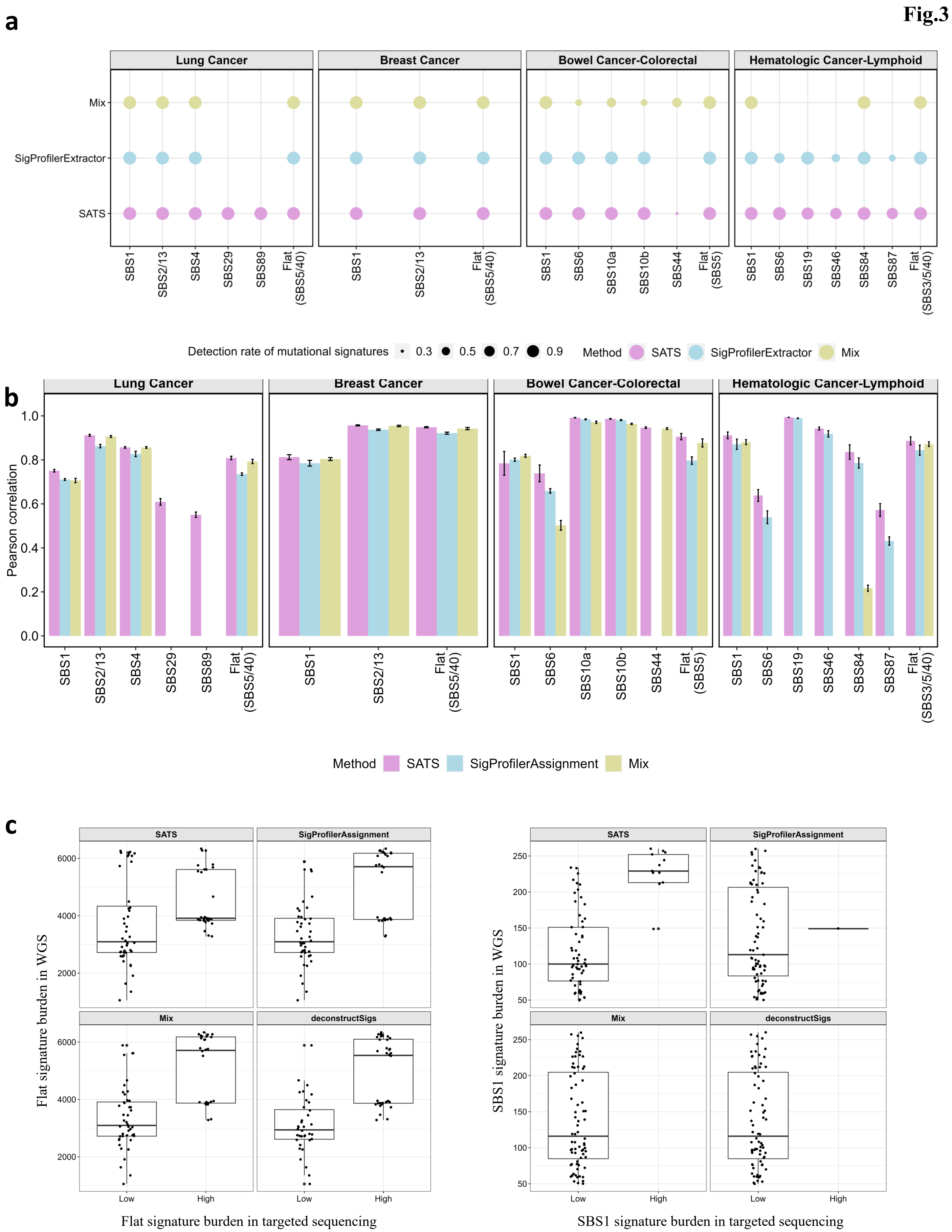

Fig.3
a
Lung Cancer
Breast Cancer
Bowel Cancer-Colorectal
Hematologic Cancer-Lymphoid
Mix
SigProfilerExtractor
SATS
SBS1
SBS2/13
SBS4
SBS29
SBS89
Flat (SBS5/40)
SBS6
SBS10a
SBS10b
SBS44
Flat (SBS5)
SBS19
SBS46
SBS84
SBS87
Flat (SBS3/5/40)
Detection rate of mutational signatures 0.3 0.5 0.7 0.9
Method SATS SigProfilerExtractor Mix
b
Pearson correlation
Method SATS SigProfilerAssignment Mix
c
SATS
SigProfilerAssignment
Mix
deconstructSigs
Flat signature burden in WGS
Low
High
Flat signature burden in targeted sequencing
SBS1 signature burden in WGS
SBS1 signature burden in targeted sequencing

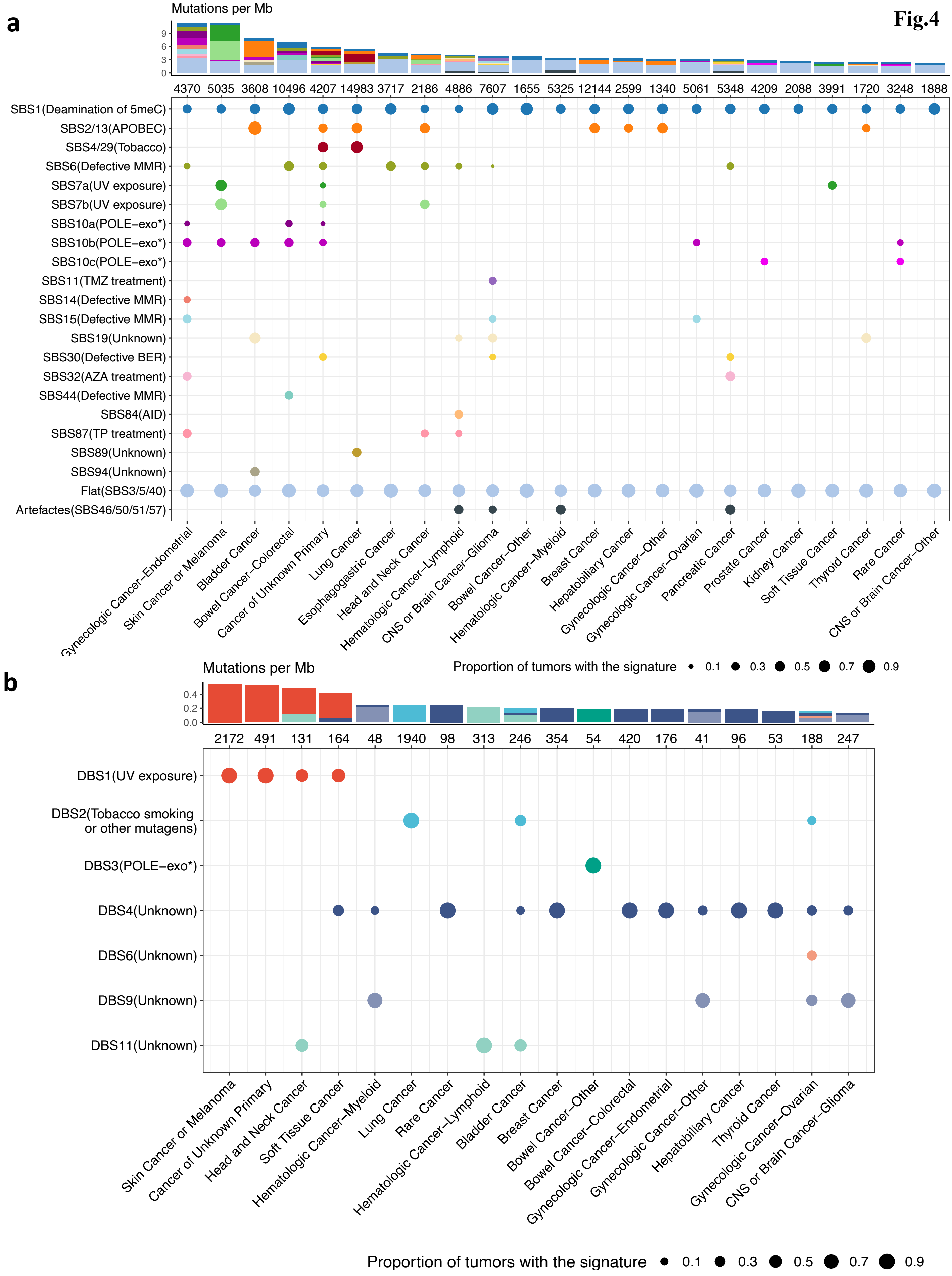

Fig.4
a
Mutations per Mb
4370 5035 3608 10496 4207 14983 3717 2186 4886 7607 1655 5325 12144 2599 1340 5061 5348 4209 2088 3991 1720 3248 1888
SBS1(Deamination of 5meC)
SBS2/13(APOBEC)
SBS4/29(Tobacco)
SBS6(Defective MMR)
SBS7a(UV exposure)
SBS7b(UV exposure)
SBS10a(POLE−exo*)
SBS10b(POLE−exo*)
SBS10c(POLE−exo*)
SBS11(TMZ treatment)
SBS14(Defective MMR)
SBS15(Defective MMR)
SBS19(Unknown)
SBS30(Defective BER)
SBS32(AZA treatment)
SBS44(Defective MMR)
SBS84(AID)
SBS87(TP treatment)
SBS89(Unknown)
SBS94(Unknown)
Flat(SBS3/5/40)
Artefactes(SBS46/50/51/57)
Gynecologic Cancer−Endometrial
Skin Cancer or Melanoma
Bladder Cancer
Bowel Cancer−Colorectal
Cancer of Unknown Primary
Lung Cancer
Esophagogastric Cancer
Head and Neck Cancer
Hematologic Cancer−Lymphoid
CNS or Brain Cancer−Glioma
Bowel Cancer−Other
Hematologic Cancer−Myeloid
Breast Cancer
Hepatobiliary Cancer
Gynecologic Cancer−Other
Gynecologic Cancer−Ovarian
Pancreatic Cancer
Prostate Cancer
Kidney Cancer
Soft Tissue Cancer
Thyroid Cancer
Rare Cancer
CNS or Brain Cancer−Other
b
Mutations per Mb
Proportion of tumors with the signature 0.1 0.3 0.5 0.7 0.9
2172 491 131 164 48 1940 98 313 246 354 54 420 176 41 96 53 188 247
DBS1(UV exposure)
DBS2(Tobacco smoking or other mutagens)
DBS3(POLE−exo*)
DBS4(Unknown)
DBS6(Unknown)
DBS9(Unknown)
DBS11(Unknown)
Skin Cancer or Melanoma
Cancer of Unknown Primary
Head and Neck Cancer
Soft Tissue Cancer
Hematologic Cancer−Myeloid
Lung Cancer
Rare Cancer
Hematologic Cancer−Lymphoid
Bladder Cancer
Breast Cancer
Bowel Cancer−Other
Bowel Cancer−Colorectal
Gynecologic Cancer−Endometrial
Gynecologic Cancer−Other
Hepatobiliary Cancer
Thyroid Cancer
Gynecologic Cancer−Ovarian
CNS or Brain Cancer−Glioma
Proportion of tumors with the signature 0.1 0.3 0.5 0.7 0.9

Fig.5

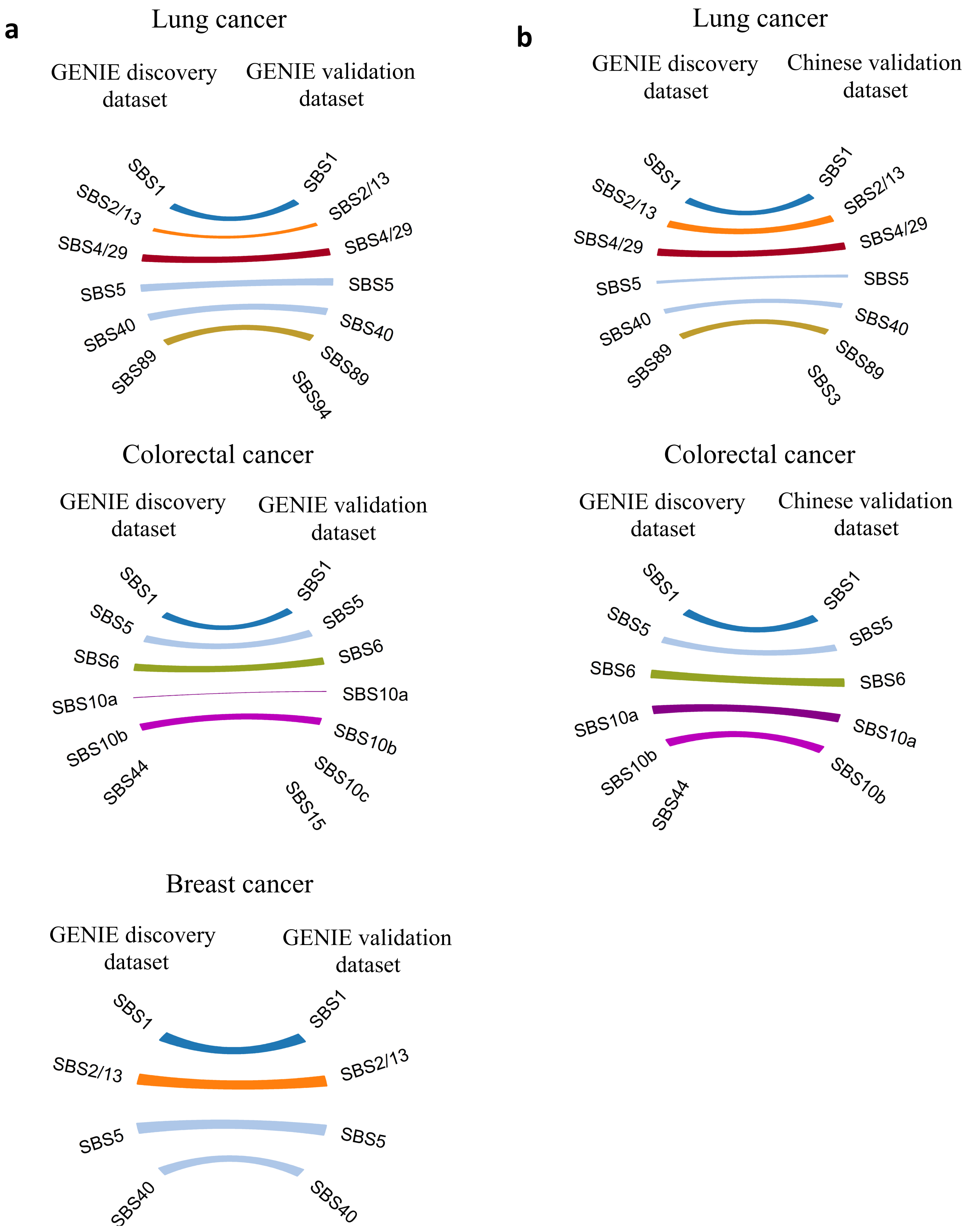

a
Lung cancer
GENIE discovery dataset
GENIE validation dataset
SBS1
SBS2/13
SBS4/29
SBS5
SBS40
SBS89
SBS1
SBS2/13
SBS4/29
SBS5
SBS40
SBS89
SBS94
Colorectal cancer
GENIE discovery dataset
GENIE validation dataset
SBS1
SBS5
SBS6
SBS10a
SBS10b
SBS44
SBS1
SBS5
SBS6
SBS10a
SBS10b
SBS10c
SBS15
Breast cancer
GENIE discovery dataset
GENIE validation dataset
SBS1
SBS2/13
SBS5
SBS40
SBS1
SBS2/13
SBS5
SBS40
b
Lung cancer
GENIE discovery dataset
Chinese validation dataset
SBS1
SBS2/13
SBS4/29
SBS5
SBS40
SBS89
SBS1
SBS2/13
SBS4/29
SBS5
SBS40
SBS89
SBS3
Colorectal cancer
GENIE discovery dataset
Chinese validation dataset
SBS1
SBS5
SBS6
SBS10a
SBS10b
SBS44
SBS1
SBS5
SBS6
SBS10a
SBS10b

Fig.6

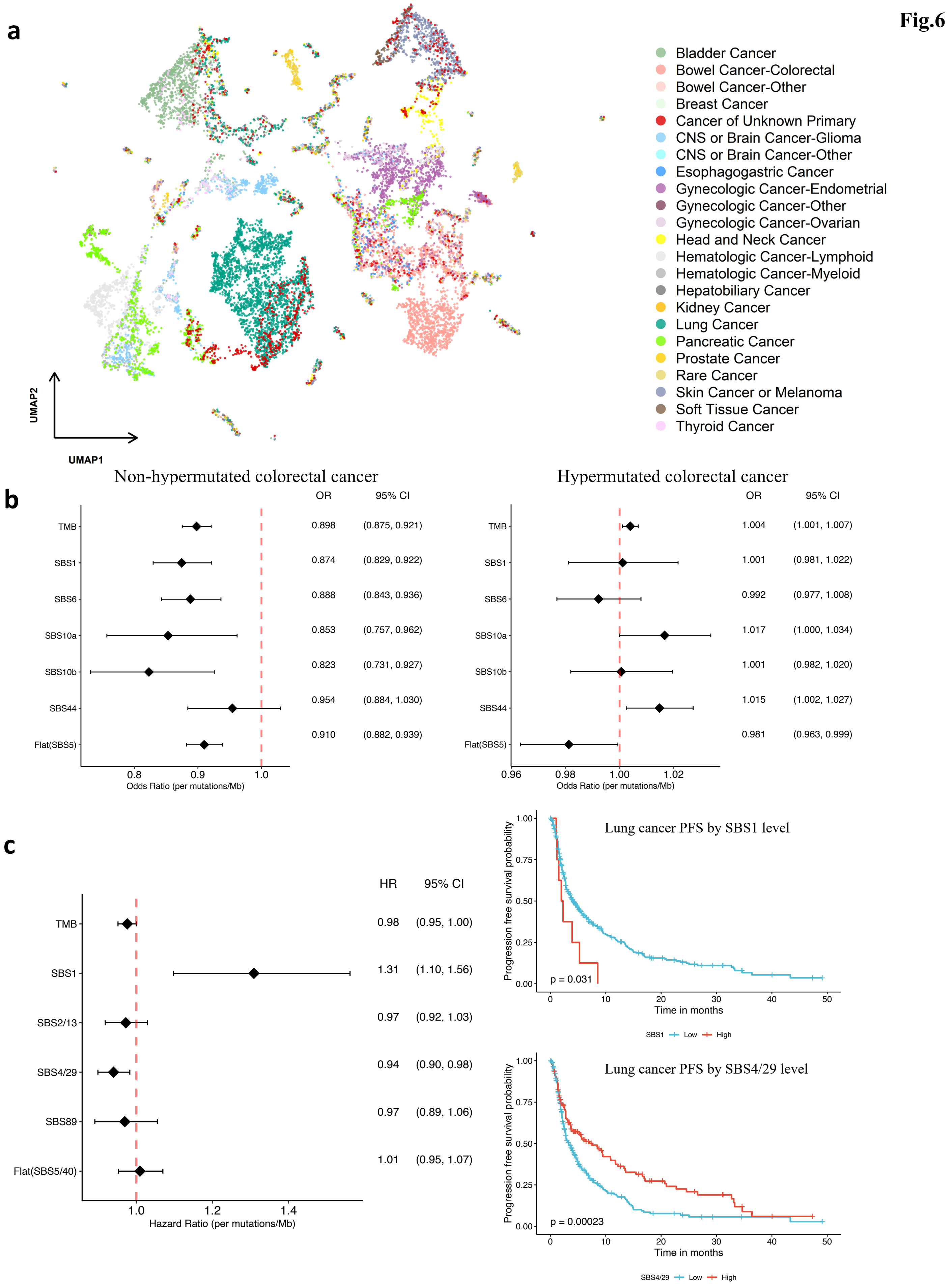

a
UMAP2
UMAP1
Bladder Cancer
Bowel Cancer-Colorectal
Bowel Cancer-Other
Breast Cancer
Cancer of Unknown Primary
CNS or Brain Cancer-Glioma
CNS or Brain Cancer-Other
Esophagogastric Cancer
Gynecologic Cancer-Endometrial
Gynecologic Cancer-Other
Gynecologic Cancer-Ovarian
Head and Neck Cancer
Hematologic Cancer-Lymphoid
Hematologic Cancer-Myeloid
Hepatobiliary Cancer
Kidney Cancer
Lung Cancer
Pancreatic Cancer
Prostate Cancer
Rare Cancer
Skin Cancer or Melanoma
Soft Tissue Cancer
Thyroid Cancer
b
Non-hypermutated colorectal cancer
OR
95% CI
TMB
SBS1
SBS6
SBS10a
SBS10b
SBS44
Flat(SBS5)
0.898 (0.875, 0.921)
0.874 (0.829, 0.922)
0.888 (0.843, 0.936)
0.853 (0.757, 0.962)
0.823 (0.731, 0.927)
0.954 (0.884, 1.030)
0.910 (0.882, 0.939)
0.8
0.9
1.0
Odds Ratio (per mutations/Mb)
Hypermutated colorectal cancer
OR
95% CI
TMB
SBS1
SBS6
SBS10a
SBS10b
SBS44
Flat(SBS5)
1.004 (1.001, 1.007)
1.001 (0.981, 1.022)
0.992 (0.977, 1.008)
1.017 (1.000, 1.034)
1.001 (0.982, 1.020)
1.015 (1.002, 1.027)
0.981 (0.963, 0.999)
0.96
0.98
1.00
1.02
Odds Ratio (per mutations/Mb)
c
HR
95% CI
TMB
SBS1
SBS2/13
SBS4/29
SBS89
Flat(SBS5/40)
0.98 (0.95, 1.00)
1.31 (1.10, 1.56)
0.97 (0.92, 1.03)
0.94 (0.90, 0.98)
0.97 (0.89, 1.06)
1.01 (0.95, 1.07)
1.0
1.2
1.4
Hazard Ratio (per mutations/Mb)
Lung cancer PFS by SBS1 level
Progression free survival probability
1.00
0.75
0.50
0.25
0.00
p = 0.031
0
10
20
30
40
50
Time in months
SBS1 Low High
Lung cancer PFS by SBS4/29 level
Progression free survival probability
1.00
0.75
0.50
0.25
0.00
p = 0.00023
0
10
20
30
40
50
Time in months
SBS4/29 Low High
