# Supplementary material for: Pan-cancer mutational signature analysis of 111,711 targeted sequenced tumors using SATS
Source: medRxiv. 2024 Apr 3:2023.05.18.23290188. Originally published 2023 May 25. Preprint. [Version 2] doi: 10.1101/2023.05.18.23290188 (PMC10327246; doi:10.1101/2023.05.18.23290188)
Supplement: Supplementary file 1 [file media-2.docx]

**Supplementary Note**

Lee et al. Pan-cancer mutational signature analysis of 111,711 targeted sequenced tumors 

using SATS

**Calculate the size of targeted sequencing panel** 

For targeted sequencing panels in AACR Project GENIE (covering a few genes to hundreds of genes, Supplementary Table 6), we calculated the number of genomic sites in which a specific mutation type of SBS or DBS could occur (Supplementary Table 3 and Supplementary Table 5 respectively). Specifically, we downloaded the genomic information of panels ([https://www.synapse.org/#!Synapse:syn26706790](https://www.synapse.org/)) and extracted the corresponding genomic sequences (from the human reference genome hg19). For SBS, we considered 96 mutation types from 32 trinucleotide mutation contexts. Indeed, the SBS was categorized into 96 SBS types composed of a mutated pyrimidine (e.g., C to G mutation) at the trinucleotide context (e.g., TCT with flanking 5’ and 3’ nucleotide), a total of 4×6×4 = 96 SBS types and $4×2×4=32$ mutation contexts. For DBS, we considered $78$ mutation types from 10 dinucleotide mutation contexts: AC, AT, CC, CG, CT, GC, TA, TC, TG, and TT. Finally, we counted the numbers of SBS or DBS mutation contexts in individual targeted sequencing panels. 

**Impact of sample sizes on SATS signature detection**

We found that identifying spiky and common signatures, such as SBS1 and SBS2/13, only requires few thousand targeted sequenced tumors (Supplementary Fig. 5b), while detecting less spiky or less common signatures needs more samples (e.g., SBS10a, Supplementary Fig. 9a). The flattest signatures SBS3 and SBS5 require a much larger number of samples, approximately 40,000 and 80,000 samples, respectively, to be detected by all panels (Supplementary Fig. 5b). Furthermore, we found that the detection probability of signature SBS44 unexpectedly started decreasing after 10,000 samples, which coincided with an increasing detection probability of signature SBS5. This indicates that when two flat signatures, SBS3 and SBS5, are detected, another relatively flat signature, SBS44, becomes difficult to detect. This observation is consistent with previous findings on mutational signature analysis of WGS data, which showed that signatures with flat profiles are likely to be misidentified as other flat signatures^1^. The remaining signatures with a prevalence of less than 5% are unlikely to be detected even with a large number of samples (Supplementary Fig. 5b), as it is for the current algorithms based on WGS and WES data^2^. Notably, the probability of detecting false positive signatures decreases from 0.35 at 10,000 samples to less than 0.01 at 200,000 samples (Supplementary Fig. 9b).

**Cancer types of AACR Project GENIE** 

AACR Project GENIE included 102 cancer types and 757 cancer subtypes defined by OncoTree^3^. To facilitate the cancer type-specific analysis, we combined 107 OncoTree cancer types into 23 analysis cancer types, such as combining “breast cancer”, “breast cancer, NOS” and “Breast Sarcoma” into breast cancer (Supplementary Table 7). Within an analysis cancer type, we aggregated cancer subtypes accounting for less than 3% of tumors as the rare group (Supplementary Table 7).

**Estimation of signature activity matrix for a subset of samples**

Given the set of signatures present in a particular cancer type, SATS can be used for signature refitting to a small number of tumor samples or even a single tumor sample. To demonstrate this, we estimated signature burdens of lung cancers in *in silico* simulations for a subset of samples at a time. 

We first generated the mutation type matrices by using the mapped signature ${\mathbf{W}}_{lung}^{\boldsymbol{*}}$ and the signature activity matrix ${\mathbf{H}}_{lung}^{\boldsymbol{*}}$ estimated from the AACR Project GENIE lung cancer study. We used the panel size matrix ${\mathbf{L}}_{lung}$ to obtain the expectation matrix ${\mathbf{E}}_{lung}^{\boldsymbol{*}}$ through element-wise multiplication, ${\mathbf{E}}_{lung}^{\boldsymbol{*}}$ = ${\mathbf{L}}_{lung}\mathbf{∘}{\mathbf{W}}_{lung}^{\boldsymbol{*}}{\mathbf{H}}_{lung}^{\boldsymbol{*}}$. We set ${\mathbf{E}}_{lung}^{\boldsymbol{*}}$ as the Poisson mean parameter and generated ${\mathbf{V}}_{lung}^{sim, 1}, ⋯, {\mathbf{V}}_{lung}^{sim, 10}$ from the Poisson distribution. We then split these datasets into submatrices with sizes 1, 10, 50, 100, 500, 1000 and 5000. With ${\mathbf{W}}_{lung}^{\boldsymbol{*}}$ fixed, we reconstructed the signature activity matrices ${\mathbf{H}}_{lung}^{sim, k}$ from the separate estimates obtained from these submatrices. Finally, we compared the true and estimated signature expectancy for various sizes.

We found that the signature burdens are consistent between the estimated and simulated ones regardless of the number of samples used (Supplementary Fig. 10). Thus, SATS provides a useful tool for analyzing individual tumors in clinical settings, by reliably estimating the signature burden for a few or even a single sample, based on a list of known signatures from targeted sequencing data.

**References**

1.	Koh, G., Degasperi, A., Zou, X., Momen, S. & Nik-Zainal, S. Mutational signatures: emerging concepts, caveats and clinical applications. *Nat Rev Cancer* **21**, 619-637 (2021).

2.	Alexandrov, L.B., Nik-Zainal, S., Wedge, D.C., Campbell, P.J. & Stratton, M.R. Deciphering signatures of mutational processes operative in human cancer. *Cell Rep* **3**, 246-59 (2013).

3.	Kundra, R. *et al.* OncoTree: A Cancer Classification System for Precision Oncology. *JCO Clin Cancer Inform* **5**, 221-230 (2021).
