# Supplementary material for: Pan-cancer mutational signature analysis of 111,711 targeted sequenced tumors using SATS
Source: medRxiv. 2024 Apr 3:2023.05.18.23290188. Originally published 2023 May 25. Preprint. [Version 2] doi: 10.1101/2023.05.18.23290188 (PMC10327246; doi:10.1101/2023.05.18.23290188)

Supplementary Fig.1

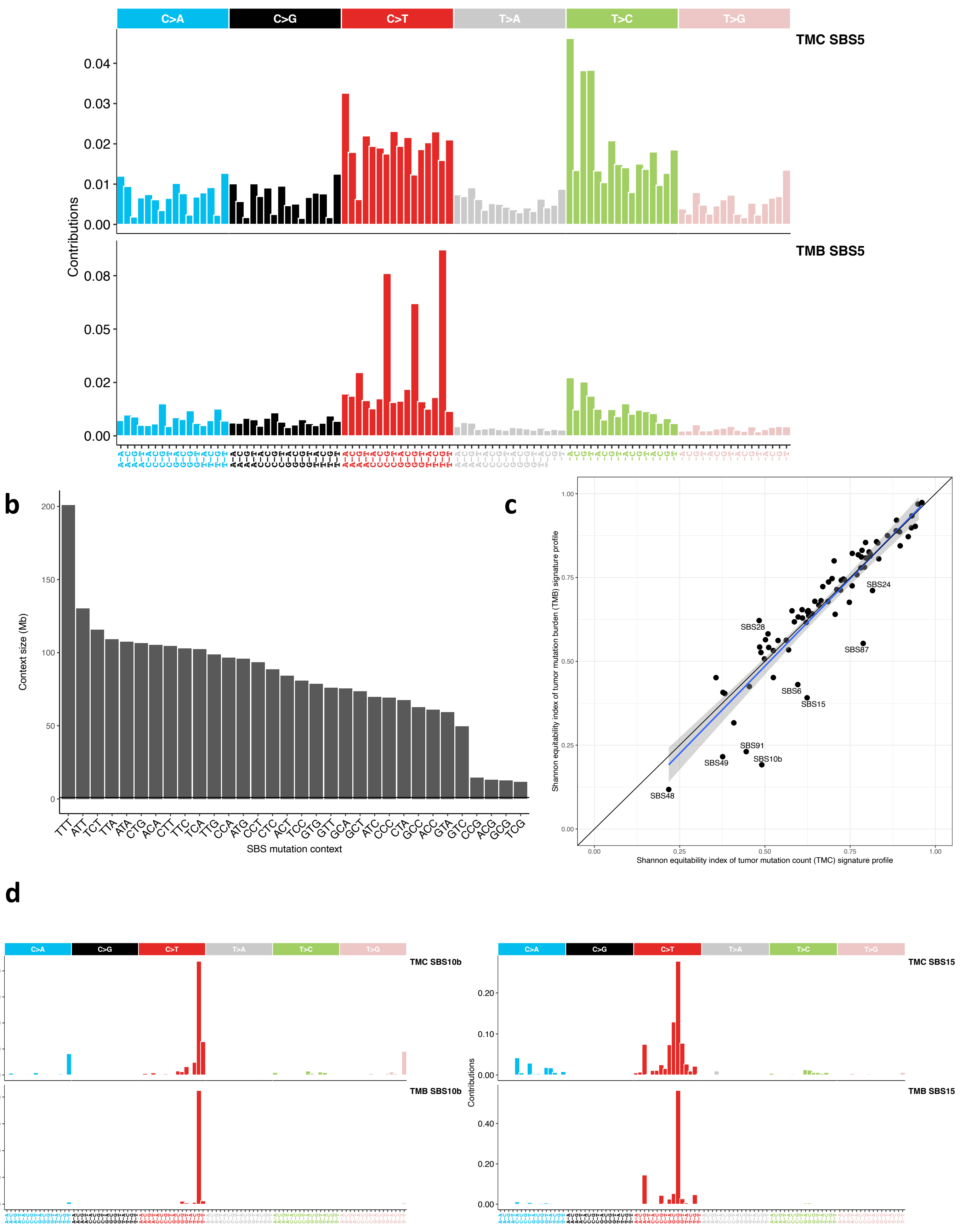

Supplementary Fig.2

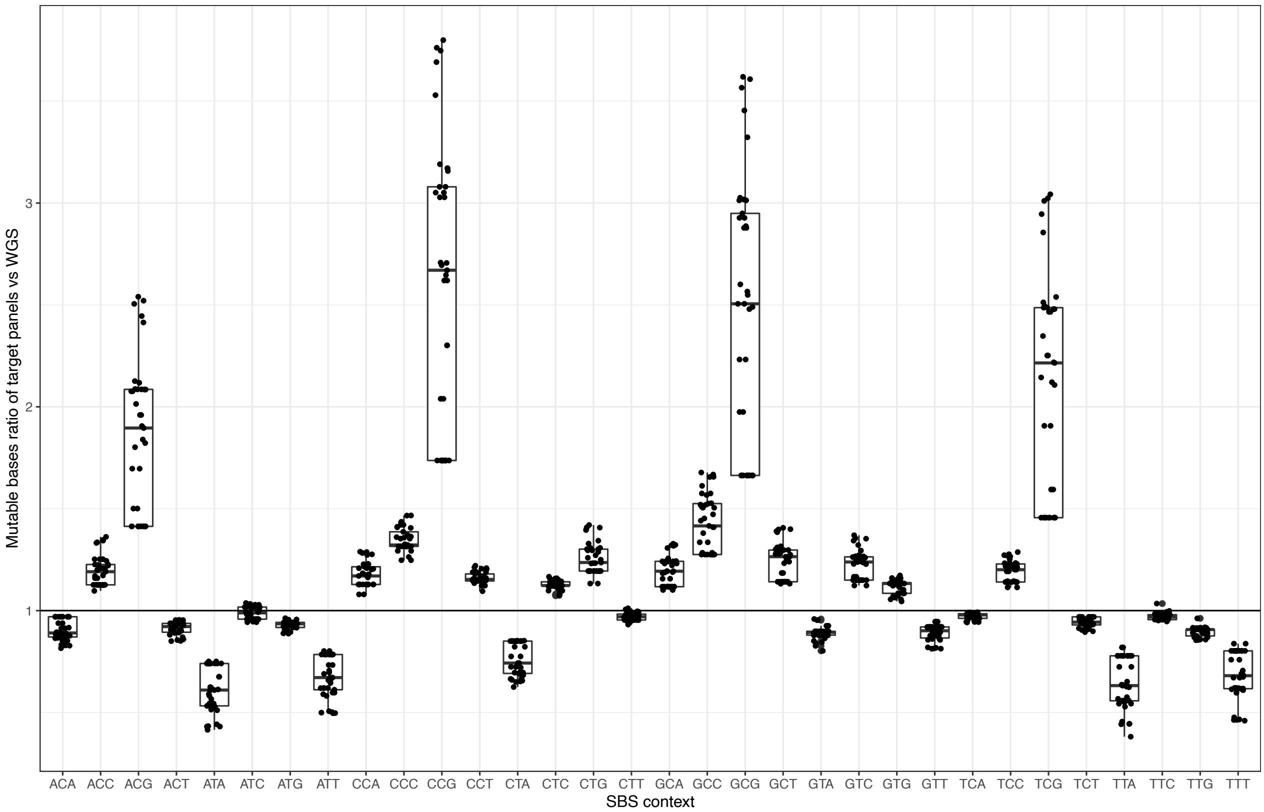

Supplementary Fig.3

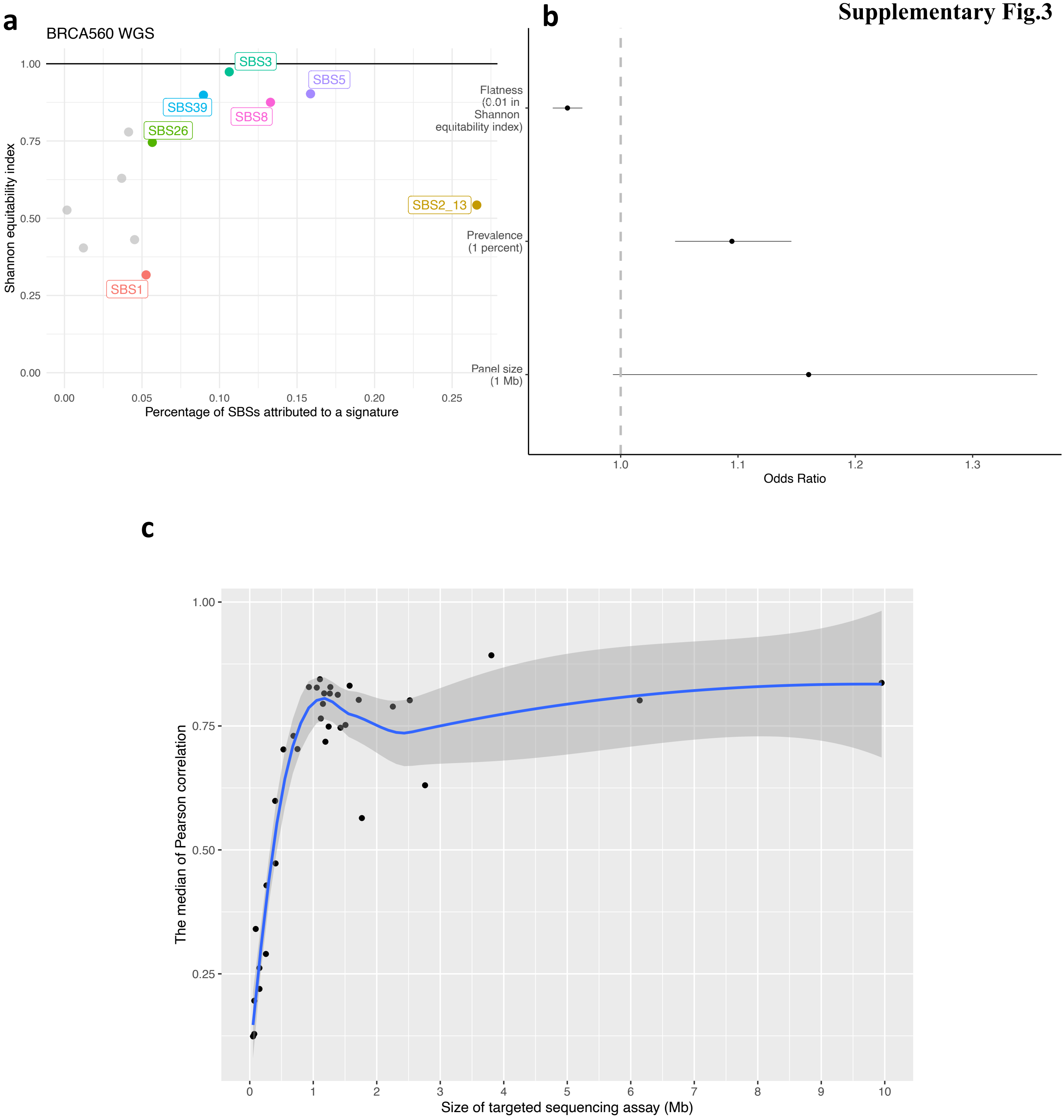

Supplementary Fig.4

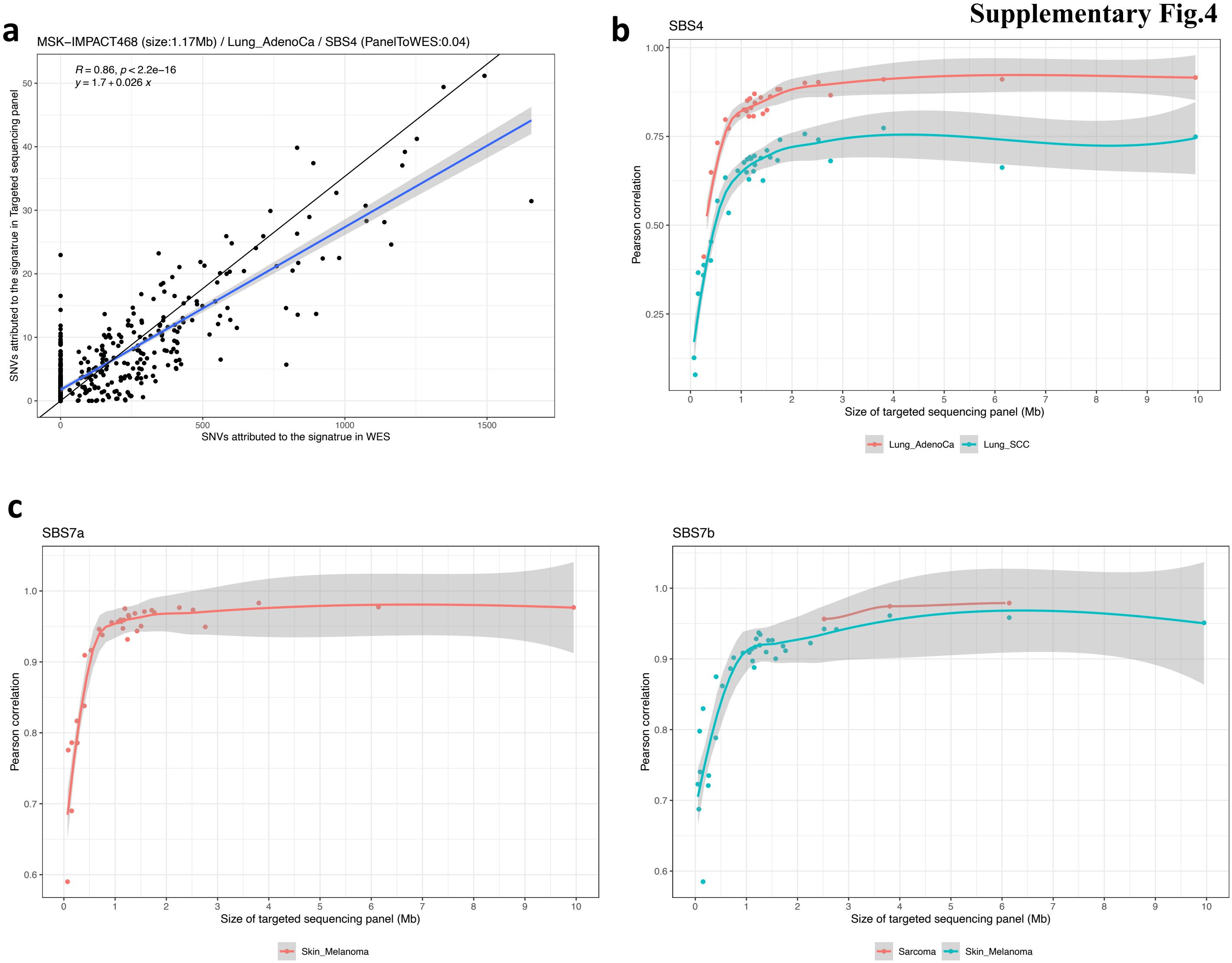

Supplementary Fig.5

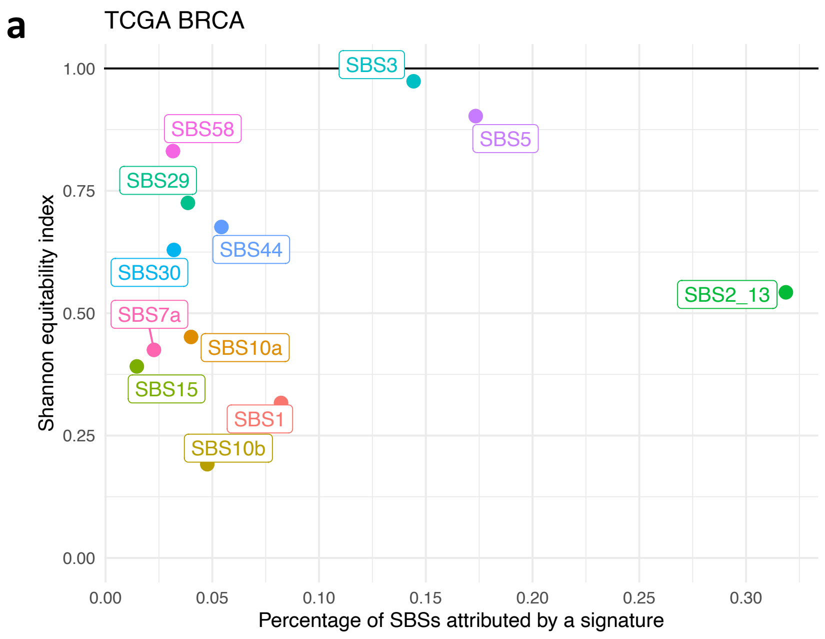

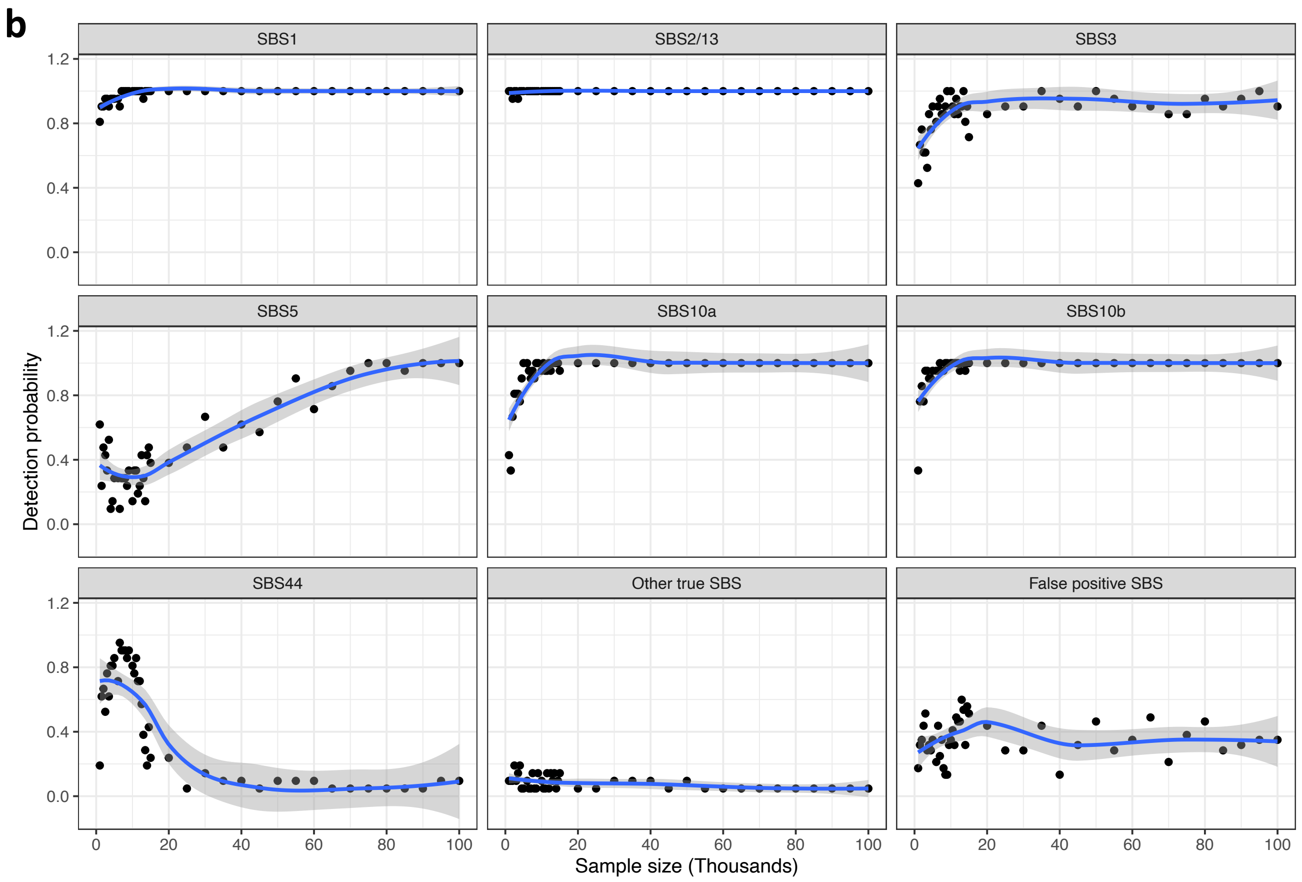

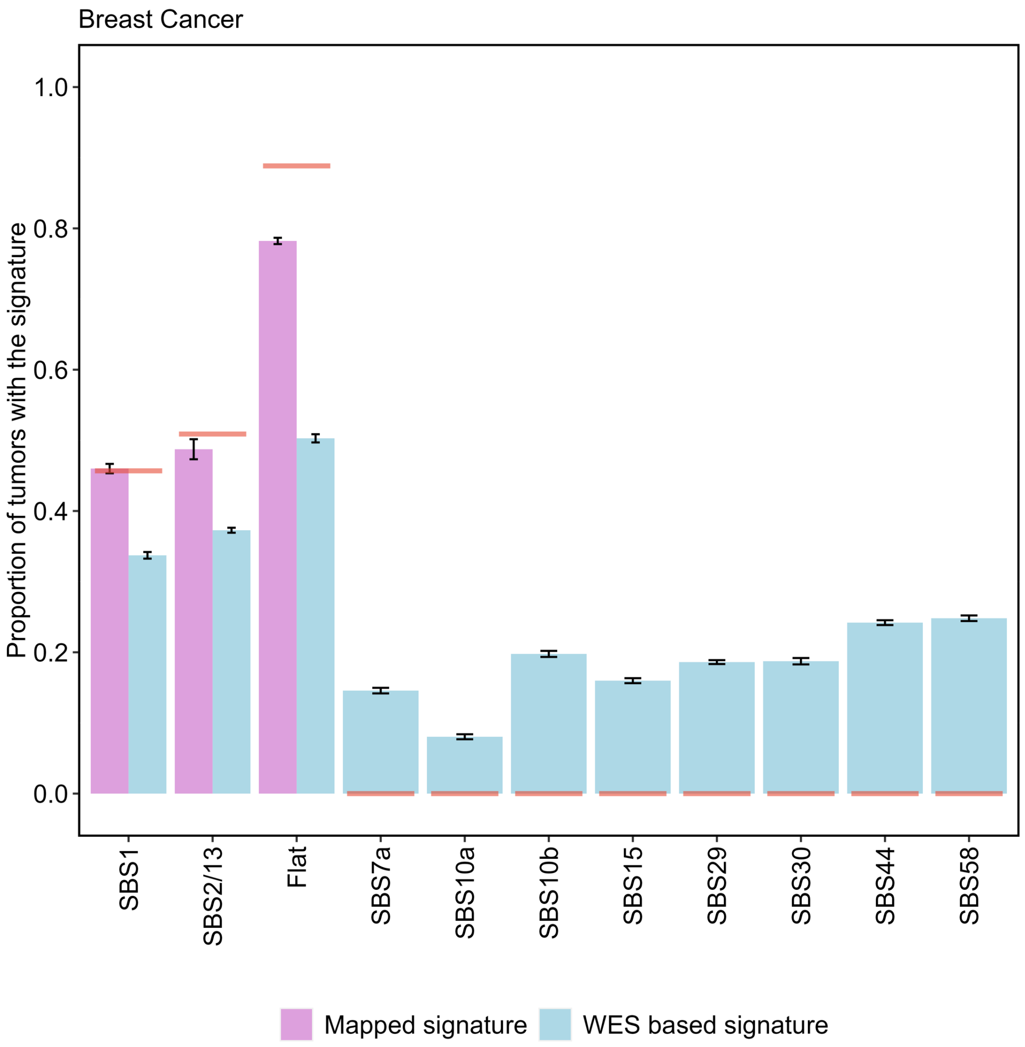
Breast Cancer
Proportion of tumors with the signature
1.0
0.8
0.6
0.4
0.2
0.0
SBS1
SBS2/13
Flat
SBS7a
SBS10a
SBS10b
SBS15
SBS29
SBS30
SBS44
SBS58
Mapped signature
WES based signature

Supplementary Fig.7

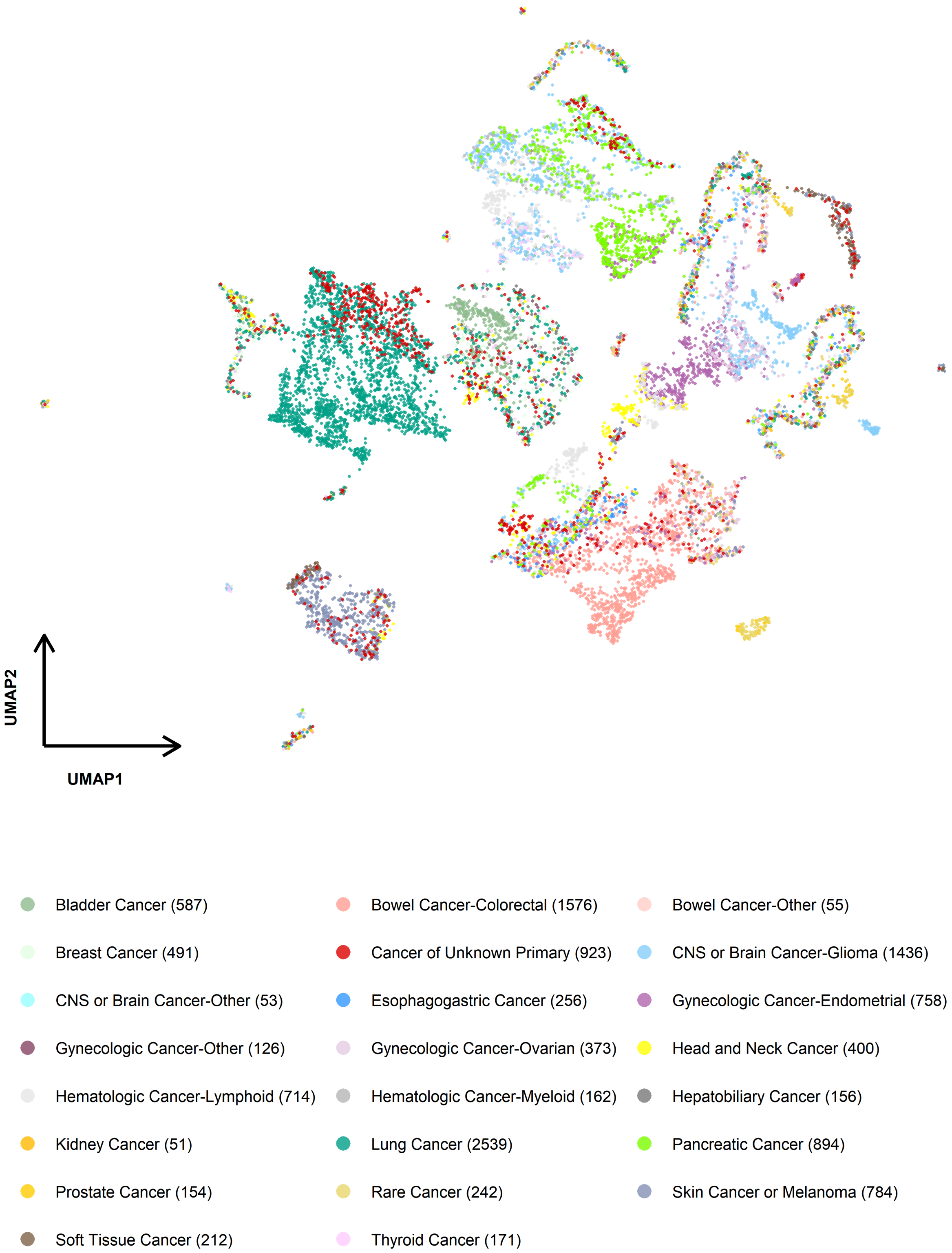

Supplementary Fig.8

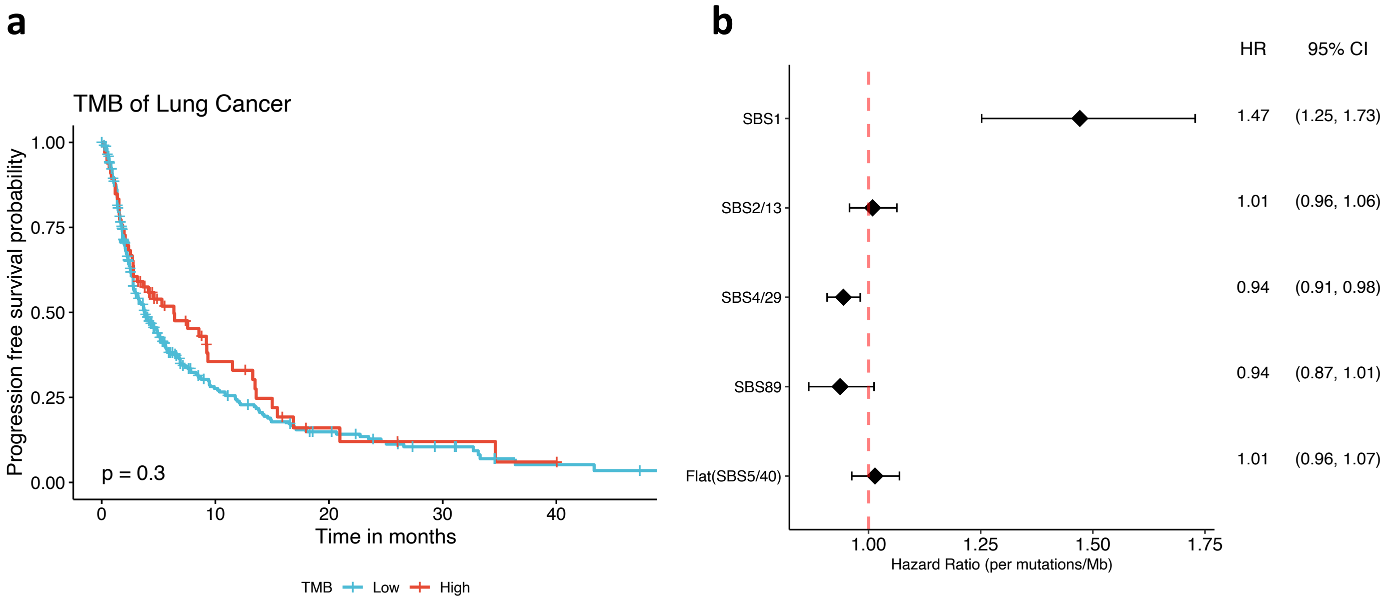

a **Supplementary Fig.9**

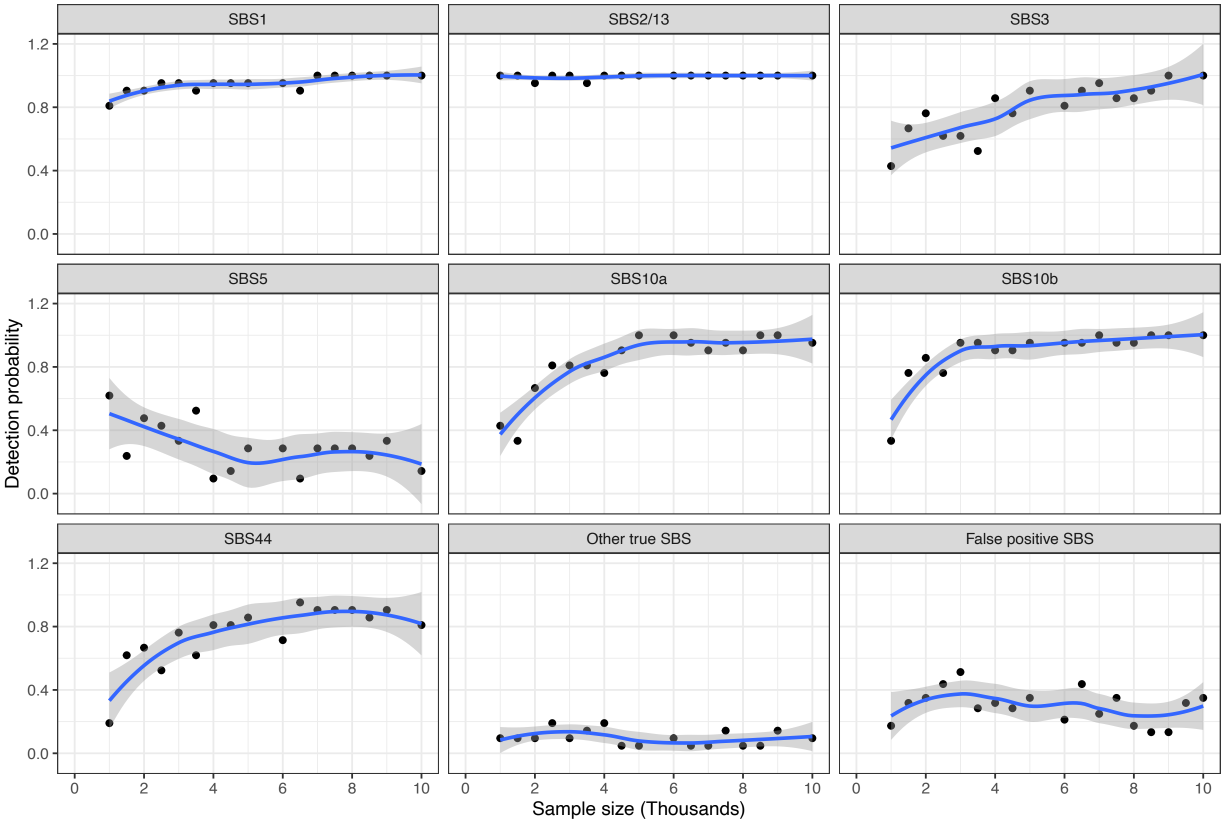

b

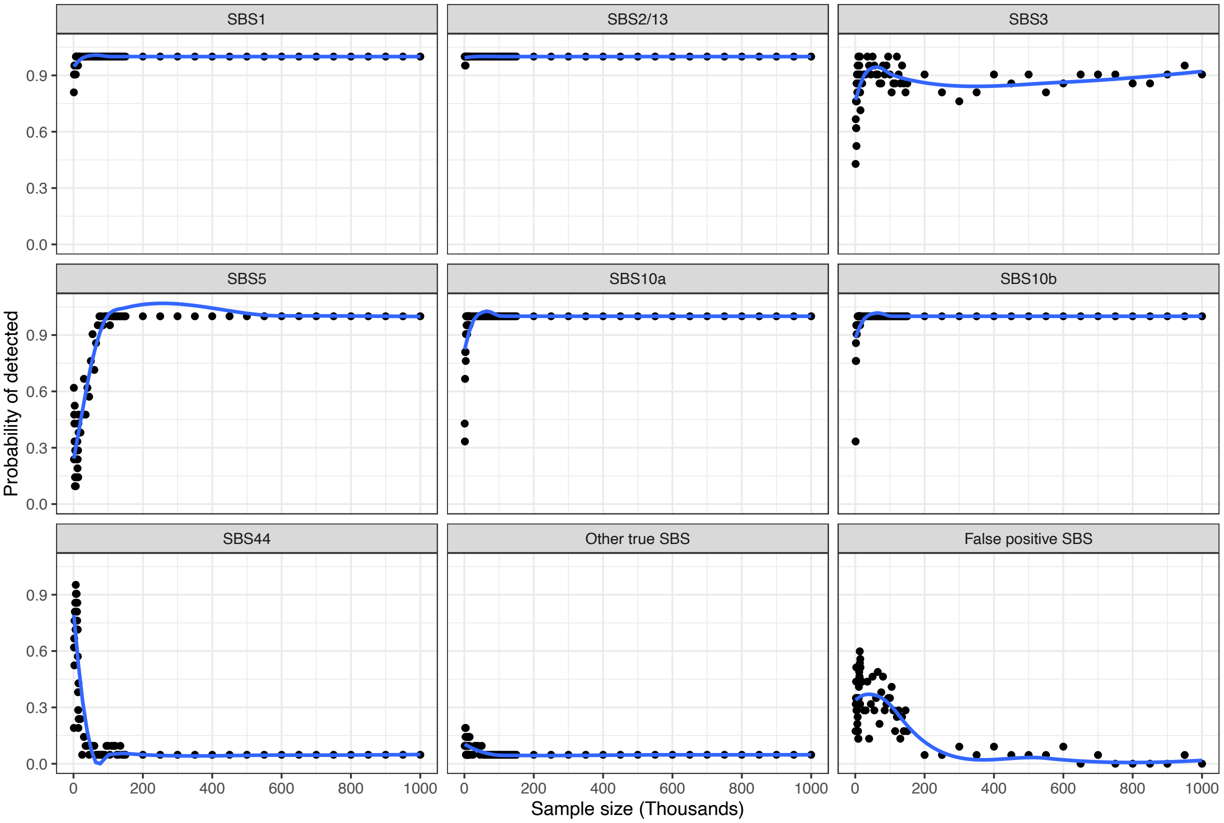

Supplementary Fig.10

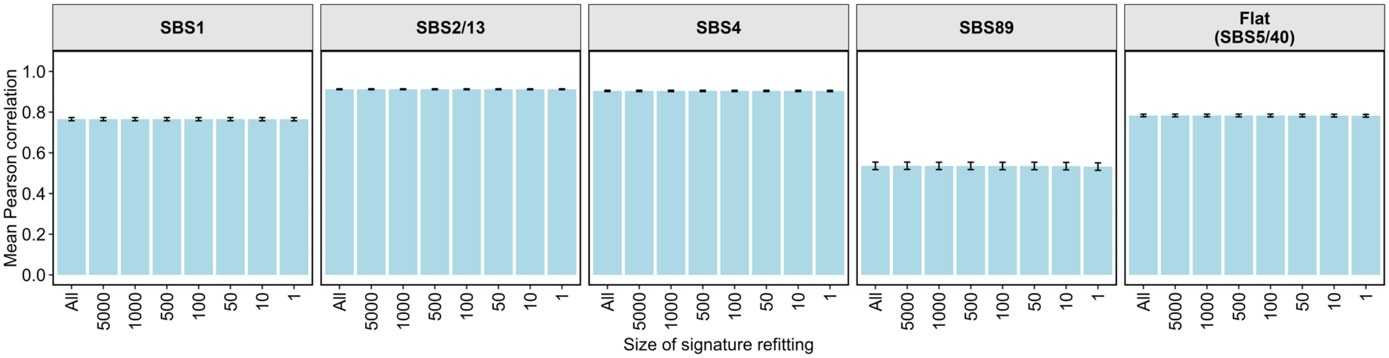

Supplement: Supplementary file 1 [file media-1.pdf]
